# Targeting signaling pathways in osteosarcoma: Mechanisms and clinical studies

**DOI:** 10.1002/mco2.308

**Published:** 2023-07-10

**Authors:** Ziyu Ji, Jianlin Shen, Yujian Lan, Qian Yi, Huan Liu

**Affiliations:** ^1^ School of Integrated Traditional Chinese and Western Medicine Southwest Medical University Luzhou Sichuan China; ^2^ Department of Orthopaedics Affiliated Hospital of Putian University Putian Fujian China; ^3^ Department of Physiology School of Basic Medical Science Southwest Medical University Luzhou Sichuan China; ^4^ Department of Orthopaedics The Affiliated Traditional Chinese Medicine Hospital of Southwest Medical University Luzhou Sichuan China

**Keywords:** clinic treatment, osteosarcoma, signaling pathway, traditional Chinese medicine

## Abstract

Osteosarcoma (OS) is a highly prevalent bone malignancy among adolescents, accounting for 40% of all primary malignant bone tumors. Neoadjuvant chemotherapy combined with limb‐preserving surgery has effectively reduced patient disability and mortality, but pulmonary metastases and OS cells' resistance to chemotherapeutic agents are pressing challenges in the clinical management of OS. There has been an urgent need to identify new biomarkers for OS to develop specific targeted therapies. Recently, the continued advancements in genomic analysis have contributed to the identification of clinically significant molecular biomarkers for diagnosing OS, acting as therapeutic targets, and predicting prognosis. Additionally, the contemporary molecular classifications have revealed that the signaling pathways, including Wnt/β‐catenin, PI3K/AKT/mTOR, JAK/STAT3, Hippo, Notch, PD‐1/PD‐L1, MAPK, and NF‐κB, have an integral role in OS onset, progression, metastasis, and treatment response. These molecular classifications and biological markers have created new avenues for more accurate OS diagnosis and relevant treatment. We herein present a review of the recent findings for the modulatory role of signaling pathways as possible biological markers and treatment targets for OS. This review also discusses current OS therapeutic approaches, including signaling pathway‐based therapies developed over the past decade. Additionally, the review covers the signaling targets involved in the curative effects of traditional Chinese medicines in the context of expression regulation of relevant genes and proteins through the signaling pathways to inhibit OS cell growth. These findings are expected to provide directions for integrating genomic, molecular, and clinical profiles to enhance OS diagnosis and treatment.

## 1 INTRODUCTION

Osteosarcoma (OS) is one of the most prevalent primary bone malignancies.^1^ In addition to chondrosarcoma and Ewing's tumor, OS, also known as osteogenic sarcoma, originates from the mesenchymal tissue^2^ and accounts for 40% of all cases of primary malignant bone tumors, with an annual incidence of one to three cases per million people.^2^ OS primarily affects adolescents, mainly in the lower femur, upper tibia, and proximal humeral epiphysis.^3^ The pathological characteristics of OS are osteoid tissue produced by spindle‐shaped mesenchymal cells, with extensive tissue heterogeneity, high local invasiveness, rapid infiltration, metastasis, and poor clinical prognosis.^4^ Although significant advances in the treatment and prognosis of OS were made in the 1970s and 1980s, there have been no breakthroughs in recent decades.^5^ Neoadjuvant chemotherapy combined with limb preservation has become the preferred clinical treatment option for OS,^6^ and combined treatment with immunotherapy, molecular targeted therapy, and traditional Chinese medicine (TCM) has also greatly reduced the morbidity and mortality of OS patients.^7^ Although the implementation of various treatment options has enhanced the 5‐year survival rate for OS patients to 60−70% without distant metastases, approximately 20−40% of patients still die from distant metastases.^8^ Moreover, the adverse effects of radiotherapy and the resistance of OS to chemotherapeutic drugs are urgent problems in the clinical treatment of OS.^9^

**FIGURE 1:**
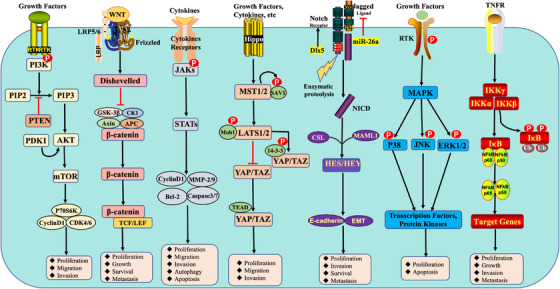
Main signaling pathways and fundamental factors in osteosarcoma. The major signaling of PI3K/AKT/mTOR, Wnt/β‐linked protein, JAK/STAT3, Hippo, PD‐1/PD‐L1, Notch, MAPK, and NF‐κB pathways were elucidated, as well as their regulatory roles in cellular processes.

Signaling pathways are signal networks wherein information is transferred between the interior and exterior regions of cells. This process includes the interaction among multiple molecules and proteins to transmit information through a series of molecules such as signaling molecules, signaling receptors, and signal transduction factors.^10^ These signaling pathways regulate cell growth, differentiation, apoptosis, metabolism, and other physiological and pathological processes.^11^ Accumulating evidence suggests that abnormal activation of signaling pathways is an important factor contributing to tumorigenesis and progression.^12^, ^13^, ^14^ In OS, the signaling pathways phosphatidylinositol 3 kinase (PI3K)/protein kinase B (AKT)/mammalian target of rapamycin (mTOR),^15^ Wnt/β‐catenin,^16^ Janus kinase (JAK)/signal transducers and activators of transcription 3 (STAT3),^17^ Hippo,^18^ programmed death‐1 (PD‐1)/programmed death ligand‐1 (PD‐L1),^19^ Notch,^20^ mitogen‐activated protein kinase (MAPK),^21^ and nuclear factor kappa B (NF‐κB)^22^ perform a fundamental function in OS development. Additionally, multiple factors including drugs, proteins, RNA, and genes either positively or negatively regulate tumor cells proliferation, invasion, and apoptosis in OS by affecting the signaling pathways JAK/STAT3, Hippo, Wnt/β‐catenin, PD‐1/PD‐L1, Notch, MAPK, PI3K/AKT/mTOR, NF‐κB, and others (Figure 1). However, identifying novel biomarkers for OS is urgently needed to develop novel and specific targeted therapies at the molecular level to improve the survival of patients with OS.

To offer a thorough overview of the recent research advances on the mechanisms and clinical investigations of OS‐targeted signaling pathways, we examine each of the OS‐related signaling pathways in this review. TCM has become a hot spot for OS treatment because of its broad action, low side effects, and multiple targeting functions.^23^, ^24^ Therefore, we have focused on the signaling targets involved in the preventive and curative effects of TCM on OS, which act by modulating the expression of relevant proteins and genes through signaling pathways to inhibit OS cells' growth, proliferation, migration, invasion, and metastasis. This review was conducted to provide ideas and discover new treatment options for OS treatment, improve patient prognosis, and increase the overall survival of patients.

## 2 SIGNALING PATHWAYS IN OS

Recently, research evidence has emerged showing that the dysregulation of various intracellular signaling pathways is closely related to the onset and progression of OS, and to treat OS, it is crucial to research the signaling pathways linked to its development. The PI3K/AKT/mTOR signaling pathway, one of the crucial pathways in cancerous cells, has been demonstrated to have the ability to enhance the cell cycle, inhibit apoptosis, and promote cellular proliferation, invasion, and metastasis. The modulation of OS cellular proliferation, apoptosis, and vascular neogenesis, among other processes, is critically controlled by the JAK/STAT3 axis, a classical intracellular signaling pathway. Numerous mammalian cells and organs are subject to homeostatic modulation by the Hippo signaling pathway, which also controls OS cellular proliferation, apoptosis, invasiveness, and metastasis. The immunosuppression and weakening of immune cell killing effect on tumor cells lead to immune escape of OS cells, consequently culminating in the occurrence and development of OS. Abnormal activation of the MARK signaling pathway leads to loss of apoptosis and differentiation of OS cells, which is intimately correlated with the malignant transformation and abnormal proliferation of OS cells. The OS's ability to proliferate, differentiate, and resist medication is influenced by the Notch signaling pathway. Furthermore, an important factor regulating OS cell proliferation, metastasis, and drug resistance is the NF‐κB pathway. This section examines the possible biomarkers for OS as well as the modulatory functions of the aforementioned signaling pathways.

### 2.1 PI3K/AKT/mTOR signaling pathway and its role in OS

#### 2.1.1 Overview of PI3K/AKT/mTOR signaling pathway

In 1984, a new signaling pathway intimately correlated with the activity of tyrosine kinase encoded by the viral oncogene was identified. In 1988, Cantley and Downes' team discovered the virus‐associated tyrosine kinase as PI3K, which generates PI3K via the mechanism of phosphorylating the 3‐OH group on the inositol ring.^25^, ^26^ This signaling pathway involved lipid kinases and was subsequently termed the PI3K signaling pathway.^27^ Further research identified AKT, mTOR, and other genes were shown to be present downstream of the PI3K signaling pathway. Thus, this pathway was subsequently named as PI3K/AKT/mTOR signaling pathway. As one of the most crucial intracellular signaling mechanisms, it regulates cellular biological processes like cellular differentiation, proliferation, and migration.^28^

The PI3K/AKT/mTOR pathway regulates various cellular functions by integrating multiple extracellular signals.^29^ PI3K is an intracellular phosphatidylinositol kinase and a key component of the cell membrane. It has both phosphatidylinositol kinase activity and serine/threonine (Ser/Thr) kinase activity.^30^ The PI3K/AKT pathway particularly involves type I PI3K, which is a heterodimer made up of the regulatory subunit (p85) and the catalytic subunit p110^31^ and is most closely related to cancer.^32^ The structural domains SH2 and SH3 found in the regulatory subunit may interact with target proteins that have matching binding sites.^33^ P85 is recruited to the adjoining plasma membrane in response to postsignaling signals from class I PI3K acceptor receptor tyrosine kinase and G‐protein‐coupled receptors. At this point, phosphatidylinositol‐4,5‐bisphosphate (PIP2) is converted to phosphatidylinositol‐3,4,5‐trisphosphate (PIP3) by the binding action of p110 to p85, thus resulting in the further activation of AKT.^34^

Phosphatase and tensin homolog (PTEN) is an antagonist of PI3K. PIP3 functions as a second messenger and binds to the intracellular signaling protein AKT through the pleckstrin homology (PH) domain. After binding, the complex undergoes conformational changes, causing AKT to translocate from the cytoplasm to the cell membrane.^35^ Consequently, phosphorylation of AKT kinase activity is enhanced by the synergistic action of phosphoinositide‐dependent protein kinase‐1 (PDK‐1) at the Thr308 site.^36^ The binding of lipid products to the PH domain of AKT promotes PDK‐1/PDK, a complex that can phosphorylate the Ser473 site of AKT.^37^ When the Thr308 and Ser473 sites of AKT are phosphorylated, AKT becomes activated, thereby promoting cell cycle progression by regulating glycogen synthase kinase 3β (GSK3β) and cyclin.^38^, ^39^

mTOR is an important Ser/Thr protein kinase present downstream of PI3K/AKT.^40^ mTOR forms two different protein complexes: mTOR C1 and mTOR C2. Activated AKT derepresses Rheb (ras homolog enriched in the brain) through its direct phosphorylation of mTORC1 or inhibition of the formation of the tuberous sclerosis complex 1/2 (TSC1/2) dimer, thereby enhancing mTORC1 activation.^41^ The eukaryotic translation initiation factor 4E‐binding protein‐1 (4EBP‐1) and ribosomal protein S6 kinase (S6K) and are two examples of downstream effectors that are phosphorylated by the activated mTORC1. However, activated S6K phosphorylates ribosomal protein S6 (rpS6) to enhance ribosomal translation efficiency.^42^ The activated mTORC1 phosphorylates the translation repressor 4EBP‐1, which attenuates its inhibitory effects on the eukaryotic translation initiation factor 4E (eIF4E) and promotes the initiation of protein translation.^43^ Therefore, The PI3K/AKT/mTOR signaling pathway is among the most critical signaling pathways in tumor cells since it stimulates cellular proliferation, invasion, and metastasis while also accelerating cell cycle and inhibiting apoptosis.^44^

#### 2.1.2 PI3K/AKT/mTOR signaling pathway and OS development and progression

In OS, the PI3K/AKT/mTOR pathway frequently experiences changes. The high frequency of PI3K mutations and/or their increased catalytic function in tumor cells is a key mechanism of proliferation and metastasis in OS.^45^ The class I PI3K p110 catalytic subunit, namely, PI3K p110a, is encoded by the PIK3CA gene. The class I PI3K “PI3K p110a” has both lipid‐like kinase and protein kinase activities, both of which contribute to AKT activation, which can phosphorylate various substrates and participate in the modulation of signaling pathways related to cellular proliferation, differentiation, migration, and apoptosis.^46^ Approximately four‐fifths of PIK3CA mutations take place in two hotspot regions: the kinase region (exon 20) and the helix region (exon 9). Mutations in these two regions not only reduce apoptosis but also promote tumor infiltration and increase the activity of the downstream kinase PI3K, contributing to the activation of the PI3K/AKT signaling pathway.^46^, ^47^ PIK3CA gene mutations increase the risk of OS aggressiveness, and LY294002, a specific inhibitor of PI3K, inhibits PI3K at the adenosine triphosphate (ATP)‐binding site activity, culminating in a decrease in p‐AKT, which is intimately linked to OS cells’ proliferation and apoptosis.^48^

The PI3K axis is primarily activated by AKT.^49^ Most OS patients have elevated expression levels of AKT and p‐AKT. Aberrant expression of p‐AKT is strongly correlated with the overexpression of PI3K and HER4, and high levels of p‐AKT are a marker of OS progression, metastasis, and poor prognosis.^50^ Zhang et al.^51^ performed the dual‐luciferase reporter gene assay and confirmed that both long‐stranded noncoding RNA (lncRNA) MSC‐AS1 and CDK6 have binding sites and can therefore bind to miR‐142. Silencing of lncRNA MSC‐AS1 enhanced miR‐142 expression, downregulated CDK6, and decreased phosphorylated protein kinase B (p‐AKT) and phosphorylated PI3K (p‐PI3K), all of which suppressed PI3K/AKT pathway activation, thereby slowing down OS progression and increasing its sensitivity to cisplatin. Silencing miR‐191‐5p upregulated protein expression of stem‐loop‐binding protein, AKT, and p‐AKT and activated the PI3K/AKT signaling pathway, consequently promoting MG63 cells proliferation, migration, and invasion.^52^

PTEN chromosomal loss (antagonist of PI3K/AKT) is a common molecule in OS.^35^ PTEN overexpression downregulated the expression of p‐AKT and p‐PI3K in OS cells.^53^ Overexpression of miR‐524,^54^ miR‐191‐5p,^52^ miR‐744,^48^ and lncRNA HULC^54^ enhanced the upregulated PTEN levels and downregulated p‐PI3K and p‐AKT levels in OS cells. Additionally, p‐AKT has an important angiogenic role in OS via the activation of vascular endothelial growth factor (VEGF)‐A.^55^

mTOR is a bridge to multiple downstream signaling pathways that could be triggered by a variety of upstream regulatory variables.^56^, ^57^ As one of the most independent components of the PI3K axis, mTOR is found at the end of the PI3K/AKT/mTOR signaling pathway.^58^ Excessive mTOR activation might result from mutations in upstream regulatory factors from several axes, including endothelial growth factor receptor, PI3K, and PTEN.^46^, ^59^, ^60^ Any disturbance in the regulation of mTOR activities can alter the modulation of OS cells’ growth and differentiation.^29^ Furthermore, relative to normal bone tissues, OS tissues have substantially greater levels of mTOR expression.^61^, ^62^ Therefore, we hypothesized that mTOR expression might be employed as a biological marker for tumor invasion and metastasis in addition to OS diagnosis. The significance of the PI3K/AKT/mTOR signaling pathway in the onset and progression of OS suggests that this signaling axis is a viable target for the treatment of cancer.

### 2.2 Wnt/β‐catenin signaling pathway

#### 2.2.1 Overview of Wnt/β‐catenin signaling pathway

The Wnt gene was discovered during the process of exploring the transcriptional mechanism of mouse mammary tumor virus and was initially named Int‐1.^63^, ^64^ Further investigation revealed that the wingless gene (from *Drosophila*) and Int‐1 were homologous and encoded the same protein, both of which were abbreviated as Wnt.^65^ Wnt signaling pathways are of two categories: classical Wnt signaling pathways and nonclassical Wnt signaling pathways.^66^ The Wnt/PCP (Wnt–c‐Jun N‐terminal kinase [JNK] pathway) and Wnt/Ca^2+^ pathway are examples of nonclassical Wnt signaling pathway.^67^ The classical Wnt signaling pathway, including Wnt/β‐catenin, is one of the most well‐studied signaling pathways.^68^

The major components of the Wnt/β‐catenin pathway include ligands (Wnt family proteins), receptors for Wnt family proteins (the primary receptor Frizzled [Fzd] protein and the coreceptor low‐density lipoprotein receptor‐related protein 5/6 [LRP‐5/6]), Dishevelled (Dsh), axin (Axin), glycogen synthase kinase 3β (GSK‐3β), casein kinase 1 (CK1), colorectal adenomatous polyposis coli (APC), and β‐linked protein (β‐catenin).^69^, ^70^ At rest, GSK‐3β, CK1, APC, and Axin form a “degradation complex,” which binds to free β‐catenin and phosphorylates it. The phosphorylated form is then ubiquitinated and degraded to maintain the cytoplasmic concentration of free β‐catenin at a relatively low level.^71^ Upon the activation of the Wnt signaling pathway, Wnt family proteins respond by binding to Fzd proteins and/or LRP‐5/6, thereby activating the pontine protein Dsh, which dissociates the β‐catenin degradation complex comprising Axin, GSK‐3β, and APC.^72^ The cytoplasmic β‐catenin is freed from the inhibition of the degradation complex and then accumulates within the cytoplasm and further enters the nucleus.^73^ DNA is not directly bound by β‐catenin when it enters the nucleus. Instead, it binds to transcriptional T‐cell factor/lymphocyte enhancer‐binding factor (TCF/LEF),^74^ which eventually initiates the transcription of cyclin D1, c‐myc, and other target genes.^75^

β‐Catenin is a key signaling protein factor in the classical Wnt signaling pathway.^76^ β‐Catenin was originally discovered as an adhesion factor, under normal physiological conditions, most of the β‐catenin molecules in the cytoplasm are bound to E‐cadherin present in the cell membrane to mediate adhesion between cells.^77^ Later on, β‐catenin was identified as a multifunctional protein with important effects on normal human physiological functions.^78^ Whether or not β‐catenin acts via the accumulation of β‐catenin signals is the main basis for distinguishing between classical and nonclassical pathways.^79^ As a critical component of the Wnt/β‐catenin signaling pathway β‐catenin is inhibited by the β‐catenin degradation complex comprising GSK‐3β, CK1, APC, and Axin and is promoted by the β‐catenin antagonistic degradation complex comprising of Dsh, CK1, and GSK‐3β.^80^ β‐Catenin expression is an important basis to determine whether there is abnormal activation of the Wnt/β‐catenin pathway.^81^

#### 2.2.2 Wnt/β‐catenin signaling pathway and OS development and progression

OS development occurs due to the activation or silencing of several signaling pathways.^82^ The signaling pathway is associated with tumorigenesis, and both Wnt3a and Wnt10b can promote metastasis of mouse lesions in OS experiments due to the activation of β‐catenin expression. Experimental results confirm that Wnt signaling can reduce patient survival by regulating the metastasis of OS cells in an autocrine or paracrine manner.^83^ In vitro experiments revealed that the activation of Wnt5a and U2OS cell lines showed significant aggressiveness and inhibition of Wnt5a inhibited OS aggressiveness.^84^ It is clinically possible to target and intervene in this signaling pathway to treat OS. Human OS samples show a high association between the expression of WNT7B/WNT9A, LOXL2, and c‐FOS, and coexpression with c‐FOS/LOXL2 was linked to OS aggressiveness and shortened patient survival duration.^85^ Syndcan2 (SDC2), a modulator of antitumor effects, inhibits SDC2 expression in the Wnt/β‐catenin pathway in OS cells, and reversal of the regulation increases SDC2 expression by secreting SFRP1, a Wnt signaling pathway antagonist, which improves OS disease progression.^86^ Secretory Fzd‐related protein 2 (sFRP2) shows high expression in patients with OS and negatively correlates with patient survival. sFRP2 overexpression induces angiogenesis and drives osteoblast precursors into the OS phenotype,^87^, ^88^ whereas sFRP2 knockdown impairs its metastatic and invasive behavior.^89^ sFRP3 blocks the paracrine microsecretory microenvironment provided by Wnt protein expression in OS cells.^90^

The positive rate of β‐catenin in OS tissues is 70%, which differs from that in paraneoplastic tissues. β‐Catenin enables the invasion of OS cells and increased β‐catenin content in the nucleus can promote the epithelial‐to‐mesenchymal transition (EMT) of OS cells and increase stem cell formation, both of which could enhance the migration and invasiveness of tumor cells as well as the growth and spread of OS cells.^91^ The development of tumor cells might be prevented by regulating the PI3K/AKT/GSK‐3β signaling pathway and inhibiting oxidative stress in the human OS SAOS‐2 cell line. The onset, progression, and metastasis of OS are all tightly linked to β‐Catenin expression in OS, which may be crucial for OS cell invasiveness and metastasis. β‐Catenin can be used as an indicator for OS diagnosis and metastasis prediction. Yang et al.^92^ demonstrated that WIF‐1 expression, a suppressor of the Wnt/β‐catenin pathway, significantly reduced in OS cells. Furthermore, in an in vitro study of OS cells, reduced expression of WIF‐1 could lead to an abnormal accumulation of the β‐catenin protein within the cytoplasm, consequently contributing to overactivation of the classical Wnt signaling pathway and promotion of cell growth, cell proliferation, and disease progression. Overexpression of Dickkopf‐3 (DKK‐3), another Wnt signaling antagonist, retards tumor growth and lung metastasis in xenograft models.^93^ LRP5 is frequently expressed in OS and is significantly associated with the chondroblast subtype and metastasis in OS.^94^, ^95^, ^96^ Similarly, another in vivo study found that dominantly inactivated LRP5 impedes the tumorigenic potential and metastasis of OS.^97^

### 2.3 JAK/STAT3 signaling pathway

#### 2.3.1 Overview of JAK/STAT3 signaling pathway

The JAK/STAT signaling pathway regulates a variety of cytokines and is crucial for immune and metabolic regulation as well as biological activities including cellular division, differentiation, and apoptosis.^98^, ^99^ The tyrosine kinase‐related receptors, the JAK family, and the STAT family make up the pathway's three main components. Some of the members of the JAK family include JAK1, JAK2, JAK3, and TYK2. Conversely, the members of the STAT family comprise seven transcription factors (TFs), namely, STAT1, STAT2, STAT3, STAT4, STAT5A, STAT5B, and STAT6,^100^ a group of over 30 transmembrane proteins that make up a superfamily. Evolutionarily, the JAK2/STAT3 signaling pathway has exhibited high evolutionary conservation and is considered the most important of all JAK/STAT signaling pathways, essentially covering the proinflammatory cytokine spectrum in human antigen‐presenting cells (APCs).^101^

Different extracellular signals can activate the JAK/STAT3 signaling pathway in vivo, mainly through the binding of extracellular cytokines (interleukin [IL]‐6, IL‐10, and IL‐11, among others) and growth factors (VEGF, fibroblast growth factor, and EGF, among others) to cell surface receptors as ligands, which dimerize the receptor molecules and induce the polymerization of intracellular glycoprotein 130 (gp130) with the α‐subunit on the receptor.^102^ The phosphorylation of tyrosine residues on the receptor is subsequently catalyzed by the activated JAKs. Further, the phosphorylated tyrosine site on the receptor and its surrounding amino acid sequence together form a mooring site in the SH2 region of the STAT3 protein.^103^ JAK facilitates the STAT3 protein's binding to the receptor when it binds to this site, resulting in the activation and phosphorylation of STAT3, which, when entering the nucleus as a dimer, binds to DNA through its DNA‐binding domain, regulating the transcription of downstream genes.^104^

#### 2.3.2 JAK/STAT3 signaling pathway and OS development and progression

The most significant STAT3 upstream protein is JAK. Additionally, the most predominant class of indirect STAT3 inhibitors that work by preventing STAT3 activation is comprised of JAK inhibitors. Only a few studies have evaluated the role of JAK in OS. In recent years, the JAK/STAT3 signaling pathway has been extensively studied, and researchers have proven that inappropriate activation of JAK/STAT3 in OS is always linked to physiological events such as defective tumor cell migration, invasiveness, metastasis, and vascular neogenesis.^105^, ^106^

OS cell lines highly express STAT3 and p‐STAT3, and inhibition of STAT3 activation can downregulate Cyclin D1 expression and further inhibit the proliferative ability of OS cells.^107^, ^108^, ^109^ The long‐chain noncoding RNA HOXD‐AS1 increases the levels of matrix metalloproteinases (MMP‐2), Bcl‐2, and Cyclin D1 through the upregulation of STAT3 and its activity, thus achieving a proproliferative effect on OS.^110^ These events promote the activation of JAK/STAT3 signaling through integrin α2β1, which triggers sustained tumor growth and cancer recurrence.^106^ In a tumor‐bearing mouse model, inhibitors of integrin α2β1 signaling significantly suppressed OS cell proliferation and the tumorigenic ability. Similarly, STAT3 also acts as a downstream effector of miR‐126,^111^ miR‐199a‐5p,^112^ miR‐19,^113^ and miR‐125b,^114^ thus regulating the proliferation of OS cells.

Couto et al.^107^ explored the apoptosis degree of four canine OS cells lines by using the small‐molecule inhibitor LLL12 to inhibit STAT3 activity and found that the apoptosis level of OS cells increased when STAT3 was inhibited, and the expression of the downstream target genes Bcl‐2, Mcl‐1, Cyclin D1, and survivin was affected by STAT3. In addition, increased apoptosis mediated by Caspase3/7 in OS cells was observed with the use of the STAT3 repressors LLL3 or S31201 or through STAT3 knockdown using small interference RNA (siRNA).^108^, ^115^

OS‐associated macrophages (tumor‐associated macrophages) promote metastasis and EMT of OS cells by elevating the expression levels of p‐STAT3,^116^ MMP‐9, and COX‐2. MSCs can secrete IL‐6 in OS cells and promote their migration and invasiveness through the production of phosphorylated STAT3, suggesting that MSCs in the normal bone tissue surrounding the OS tissue can undergo OS metastasis and inhibit apoptosis of tumor cells through the STAT3 pathway.^117^ RNA‐Seq assay has shown that knockdown of KDM5A induces OS cells apoptosis through the IL‐6/JAK/STAT3 pathway.^118^ Using microarray and bioinformatics analysis, Lv et al.^119^ found that serglycin promotes OS cell proliferation, migration, and invasion through the JAK/STAT signaling pathway.

Metastatic OS involves the formation of a very complex and robust vascular system through angiogenesis. More specifically, metastatic OS cells can form tumor blood vessels directly by mimicking angiogenesis without relying on the involvement of endothelial cells.^120^ Exogenous administration of tumor suppressor M, belonging to the IL‐6 cytokine family expressed in canine and human OS cells, promotes phosphorylation of STAT3 and upregulates Src and JAK2 activity, significantly increases VEGF expression in OS cells, and enhances OS neovascularization through STAT3‐specific inhibition of LLL3, which inhibited STAT3 activation and tumor angiogenesis induced by tumor suppressor M.^121^

### 2.4 Hippo signaling pathway

#### 2.4.1 Overview of Hippo signaling pathway

When the *Drosophila* hippo gene was discovered in 2003, Pan and Hariharan's group discovered that Hippo inactivation regulated tissue development in a negative manner. This brought attention to the Hippo signaling pathway.^122^ Subsequent research revealed that the evolutionarily highly conserved Hippo signaling pathway involves a junction protein and a repressive kinase that regulates Yorkie (a growth‐promoting transcriptional regulator).^17^ In mammals, the Hippo signaling pathway is responsible for signal transmission from the cytoplasm and plasma membrane to the nucleus, whereby it modulates the expression of multiple different target genes to modulate cell proliferation, differentiation, and apoptosis.^123^

Typical Hippo signaling pathways include the mammalian STE20‐like protein kinase 1/2 (MST1/2, a *Drosophila* Hippo homolog), transcriptional coactivator with PDZ‐binding motif (TAZ), Yes‐associated protein 1 (YAP), Mob kinase activator 1 (MOB1), Salvador homolog 1 (SAV1), and large tumor suppressor 1/2 (Lats1/2).^122^ Upon the activation of the Hippo pathway, MST1/2 kinase is phosphorylated to activate Lats1/2 kinase which subsequently stimulates MOB1 with the assistance of Sav1. Thereafter, YAP/TAZ is phosphorylated through the interaction of the Lats1/2 PPxY (PY) motif with the WW domain of YAP/TAZ. Following phosphorylation, YAP/TAZ localizes in the cytoplasm after binding to 14‐3‐3 where it is destroyed by the β‐transducin repeat‐containing E3 ubiquitin‐protein ligase‐dependent proteasome and before being lost via transcriptional coactivation. Once the Hippo pathway is suppressed, YAP/TAZ translocates to the nucleus and it interacts with the TEA domain‐containing sequence (TEADs) and other specific TFs to activate downstream target genes and promote tumor formation.^124^ With dysregulation of this pathway, TAZ and YAP oncoproteins, in particular, are often expressed at a high level in OS, suggesting the involvement of the Hippo pathway in OS development.^125^

#### 2.4.2 Hippo signaling pathway and OS development and progression

In OS, the dysregulation of the Hippo signaling pathway may depend on Sox‐2 levels.^126^, ^127^ Basilico showed that Sox2 inhibits two Hippo activators in OS, Nf2 (merlin) and WWC1 (kibra), thus blocking the Hippo pathway. Inhibiting Nf2 and WWC1 induces YAP expression and increases the tumorigenicity of OS.^126^ Currently, the same research team validated these results in Sox2 conditional knockout animals.^127^ The peroxisome proliferator‐activated receptor‐γ agonist thiazolidinedione (TZD) was also used to illustrate the crucial function of Sox‐2 in promoting OS progression. Only through YAP chelation in the cytoplasm does TZD influence OS cellular proliferation in populations of carcinoma cells that highly express SOX‐2.^128^

The TF TEAD1 is implicated in YAP‐driven OS progression transcriptionally. The crucial function of TEAD in the proliferation of YAP‐driven OS cell lines has been shown using knockdown methods. For instance, the YAP1/TEAD1 transcriptional complex was discovered to be a significant dysregulated pathway of Hippo signaling in OS using a knockdown method.^129^ USP1 deletion reduced the accumulation of TAZ in the nucleus, inhibited the interaction of TAZ with the TEAD TFs, and decreased the expression of downstream genes involved in the Hippo signaling pathway (CYR61, RUNX2, and c‐Myc).^130^ Posttranscriptional or posttransduction changes regulate the expression of YAP in OS. Recent research has shown that several miRNAs perform a carcinogenic function in OS, enhancing the expression and activity of YAP. As an illustration, miR‐375 ^131^ enhances YAP1 activity and miR‐624‐5p induces OS cell proliferation, migration, and invasive capacities by elevating the number of nuclear YAPs.^132^

### 2.5 PD‐1/PD‐L1 signaling pathway

#### 2.5.1 Overview of PD‐1 /PD‐L1 signaling pathway

The expression of PD‐1 is evident in immunocytes like tumor‐infiltrating lymphocytes (TIL), dendritic cells (DCs), monocytes, B cells, and T cells. PD‐1/PD‐L1 mediates immune responses through T cells: APCs and tumor cells both express PD‐L1 and T‐cell dysfunction and even failure may occur when PD‐1 binds to PD‐L1 in T cells. PD‐L1 overexpression in tumors allows self‐escape from cytotoxic T cells (CD8+)‐mediated cell killing.^133^ APCs and activated T cells both produce the protein B7‐1 (CD80), which binds to PD‐L1 in cancerous cells to weakly activate effector T cells.^134^ Treg CD4+, a different subtype of T cell, maintains PD‐1 expression on its surface to provide a highly immunosuppressive tumor milieu. The PD‐1 protein of Treg cells promotes the conversion of primary CD4+ T cells to Treg cells, thereby suppressing the immune response, particularly by repressing the mTOR‐AKT signaling and increasing the Treg expression and immunosuppressive function of CD4+ T cells.^135^ As seen above, PD‐1 expression not only attenuates the immune‐promoting effect of effector T cells but also enhances the suppressive effect of immunosuppressive Treg cells.

The steps of the antitumor immune cycle include the release of tumor antigens (Ags), Ag presentation, trigger and activation, T‐cell trafficking to the tumor, T‐cell infiltration, T‐cell recognition of cancer cells, and the killing of malignant cells. PD‐1/PD‐L1 affects immunity mainly by impairing the activity of cytolytic T cells.^136^ Clinical mAb‐based immunotherapy has significant therapeutic effects. APCs are released from cancer cells and present Ag to trigger T cell activation. During this process, the T cells first provide an activation signal through the T‐cell receptor (TCR) containing the Ag peptide complex presented by the major histocompatibility complex (MHC). Then, the TCR activates the signaling pathway resulting in the activation of T‐cells together with costimulation signals of the CD86/CD28 axis. Although it binds to CD86, the cytotoxic T lymphocyte‐associated protein 4 (CTLA‐4) inactivates T cells. When PD‐L1 is present on APC or tumor cells, PD‐1, which is produced on activated T cells, binds to it to dampen immunity. Thus, antitumor immunity is improved by specific blockade of the mAb‐mediated PD‐1/PD‐L1 pathway.^137^

#### 2.5.2 PD‐1/PD‐L1 signaling pathway and OS onset and progression

The level of PD‐1/PD‐L1 expression correlates with the prognosis of tumors and is substantially elevated in OS tissues. PD‐1 and PD‐L1 were found to be expressed in 47 and 53% of biopsy specimens, respectively, according to studies examining PD‐1, PD‐L1, and associated immunomarkers in samples of individuals with OS.^138^ Additionally, a negative correlation exists between PD‐1/PD‐L1 overexpression and patient survival.^139^, ^140^, ^141^, ^142^ Consequently, PD‐1/PD‐L1 overexpression is a poor prognostic indicator for OS.

**FIGURE 2:**
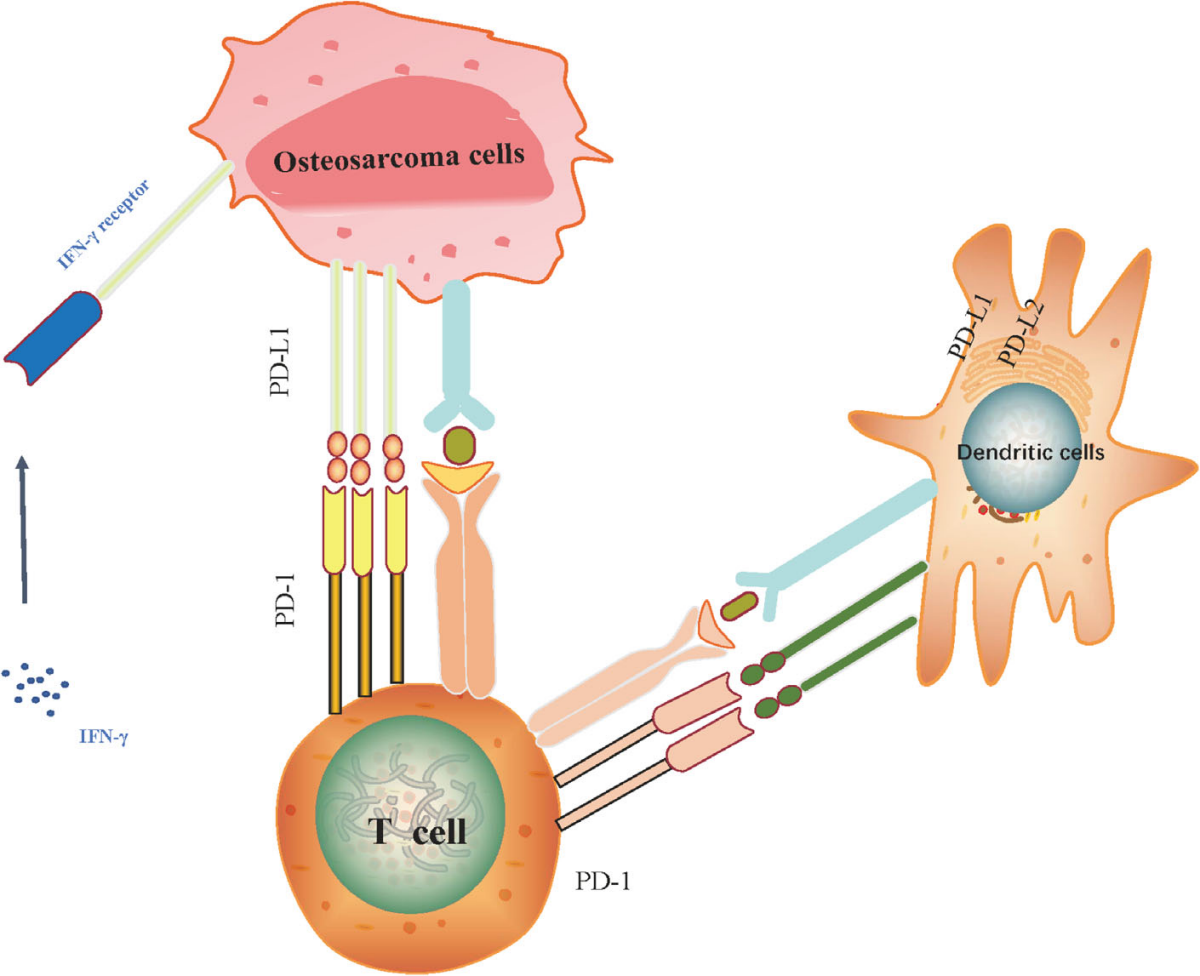
Mechanism of the PD‐1/PD‐L1 signaling pathway in OS. The immune checkpoint proteins PD‐1 on the surface of T cells interact with the ligands PD‐L1/PD‐L2 on OS cells, IFN‐γ produced by T lymphocytes induces PD‐L1 expression in angiosarcoma, resulting in immune tolerance.

When OS cells exhibit high immunogenicity, follicular helper T cells overexpress PD‐1 and underexpress the antitumor substance IL‐21, thereby attenuating the ability of CD8+ T cells to release interferon‐γ (IFN‐γ) and degranulation, which, in turn, reduces the toxicity of T cells to OS cells.^143^ IFN‐γ is produced by mitogen‐stimulated T lymphocytes, a highly effective antiviral bioactive substance that plays a role in tumor immunity.^144^ IFN‐γ was found to induce PD‐L1 expression and consequently immune tolerance in angiosarcoma^145^ (Figure 2). Yoshida et al.^146^ observed this phenomenon in OS and reported that anti‐PD‐L1 antibody significantly reduced Foxp3+ regulatory cells (Tregs) in CD4+ T cells but increased the number of Tregs and CD8+ T‐cell infiltration,^147^ which, in turn, exerted antitumor effects. Markel et al.^148^ showed that anti‐PD‐L1 antibodies declined T‐cell depletion, suggesting that T cells perform an instrumental function in the PD‐L1‐mediated development of immune tolerance in OS. PD‐L1 is upregulated in the MG63 cell line, and this cell line is tolerant to the killing effect of natural killer (NK) cells. When PD‐1/PD‐L1 is blocked, the cytotoxicity of NK cells is enhanced, which then exerts the killing effect on OS cells through granzyme B (GZMB).^149^ Yoshida et al.^150^ studied 19 clinical specimens of OS and reported a significant and positive association of IFN‐γ and GZMB with PD‐L1. The increasing infiltration of CD4+ and CD8+ T cells at the primary site of OS and the correspondingly enhanced PD‐L1 expression, and IFN‐γ stimulation contributed to the upregulation of PD‐L1 in OS cells lines, suggesting that OS can adapt to immune resistance and produce PD‐L1, which, in turn, evades the killing effect of immune cells.^151^ In summary, by weakening CD4+ T cells, CD8+ T cells, and NK cells, PD‐1/PD‐L1 could trigger the immune escape of OS cells.

PD‐1/PD‐L1 trans‐signaling pathways mediating the immune escape of tumor cells in OS involve relevant signal transduction mechanisms, which have received much attention from researchers. Blocking STAT3 elevated the expression levels of DCs that are triggered by the innate immune system, enhances the production of chemokines and cytokines in tumor tissues, triggers tumor T‐cell responses,^152^, ^153^ and mediates the body's immune response. STAT3 regulates PD‐L1 expression.^154^ Dhupkar et al.^155^ observed PD‐L1 expression in both primary and metastatic OS in humans and found that anti‐PD‐L1 antibodies blocked p‐STAT‐3/p‐extracellular signal‐regulated kinase (ERK)1/2 signaling, activated M1 macrophages, and decreased M2 macrophage numbers, resulting in a considerable decrease of OS lung metastatic sites. Histone deacetylase 6 (HDAC6) promotes PD‐L1 upregulation in OS cells, and its mechanism of action in PD‐L1 modulation is primarily driven by STAT3, while selective HDAC6 inhibitors significantly inhibit OS growth.^156^ PTEN protein inhibits PD‐L1 expression in cells.^157^ FYVE, RhoGEF, and PH structural domain‐containing protein 1 (FGD1) may bind to the N‐terminal region of PTEN,^158^ thereby suppressing PTEN function, which subsequently promotes PI3K/AKT activation, PD‐L1 upregulation, and OS progression.^159^ Additionally, high expression of myeloid‐derived suppressor cells (MDSCs) in OS can remodel the tumor microenvironment (TME) to promote tumor progression, while PI3Kδ/γ is highly expressed in MDSCs, and application of PI3Kδ/γ inhibitors induces CD8+ T‐cell infiltration and upregulation of PD‐L1 levels in OS cells.^159^, ^160^

miRNAs are closely associated with PD‐L1 expression.^161^ miR‐140,^162^ which is weakly expressed in OS, binds to the 3′‐UTR of the downstream targeted gene mRNA and directly downregulates PD‐L1 levels, increases cytotoxic CD8+ T‐cell infiltration, and decreases Tregs expression, which, in turn, inhibits the growth of OS cells, an effect that could be achieved by suppressing the mTOR/S6Ks signaling pathway. miRNA‐200a^163^ is involved in OS progression, and its enhanced expression in OS is significantly linked to unfavorable prognosis in OS. miRNA‐200a upregulates PD‐L1 protein levels, reduces the number of CD8+ and CD4+ T cells, and increases the proportion of Foxp3+ Tregs, both in vivo and in vitro. Additionally, PTEN inhibits IFN‐γ secretion by cytotoxic T lymphocytes, thus promoting the growth of OS. miR‐106a interacts with lncRNA (LINC00657) and upregulates PD‐L1 expression, which enhances OS invasion.^164^

### 2.6 Notch signaling pathway

#### 2.6.1 Overview of Notch signaling pathway

There are two types of Notch signaling pathways: classical and nonclassical. The classical Notch signaling pathway mainly includes Jagged/Jagged ligands (Jagged 1 and Jagged 2), Delta‐like ligands (DLL1, DLL3, and DLL4), receptors (Notch 1, Notch 2, Notch 3, and Notch 4), as well as TF C‐promoter‐binding factor (CBF), and its coactivators.^165^ The nonclassical Notch signaling pathway either triggers a cascade through a set of nonclassical ligands or activates target genes downstream of the nonclassical Notch signaling pathway. Unlike the Delta‐like ligands and serrate ligands, these nonclassical ligands lack the Delta/serrate/Lag‐2 domains and could be classified into three categories: secreted proteins, membrane proteins anchored to glycosylphosphatidylinositol, and integral membrane proteins.^166^ In signal‐receiving cells, receptor protein hydrolysis is triggered by the binding of Notch ligands to receptors. Two further proteolytic cleavages are mediated by the enzymes γ‐secretase and a disintegrin and metalloprotease (ADAM): one in the Notch receptor's transmembrane (ADAM) and the other in the Notch intracellular domain (NICD) from the extracellular domain (γ‐secretase).^167^ The NICD activates downstream target genes by stimulating the TF CBF1.^168^ Hairy/enhancer‐of‐split 1 (HES1) and YRPW motif‐related hairy/enhancer‐of‐split related with YRPW motif 1 (HEY1) constitute the most significant genes within the Notch pathway. In the Notch pathway, HEY1 is a crucial downstream target gene that can influence tumor cells' fate through the Notch/HES1/HEY1 pathway.^169^ In malignancies, the Notch signaling pathway is activated. Direct cell‐to‐cell interface is facilitated by its abnormal activation, which also performs a remarkable function in tumor onset, progression survival, proliferation, invasion, and metastasis.^170^

#### 2.6.2 Notch signaling pathway and OS development and progression

Cell migration‐inducible protein (CEMIP) exerts oncogenic effects in several cancers.^171^, ^172^, ^173^ CEMIP promotes OS cell growth and metastasis by activating the Notch pathway, and silencing of CEMIP in vitro and in vivo decreases the protein expression as well as the stimulation of the Notch/Jagged1/Hes1 signaling pathway.^174^ Jagged 1, an important ligand of Notch, is an integral part required to activate the Notch pathway through its binding to the Notch1 receptor and performs a fundamental function in tumor cell cycle regulation.^175^, ^176^ High expression of the lncRNA MEG3 in OS cells substantially lowered the levels of Jagged 1 and Notch 1 and restricted cell proliferation.^177^ miR‐26a directly inhibits the Jagged 1 ligand, thus suppressing the activation of the Notch pathway to resist OS proliferation.^178^ The TF Dlx5 is expressed during the initial stages of skeletal development and could assume an integral function in controlling the osteogenic process.^179^ The findings from dual‐luciferase reporter gene assay and bioinformatics analysis have suggested that Dlx5 may interact directly with Notch 1 to control Notch 1 transcription and the expression of downstream target genes, thereby promoting OS cell proliferation.^180^

The Notch signaling pathway affects tumor drug resistance by reducing intracellular drug accumulation and regulating EMT, leading to miRNA dysregulation, apoptosis gene disruption, and regulating tumor stem cells.^181^ The expression levels of Notch‐ICN, MRP1, and other proteins were remarkably elevated in hypoxia relative to normoxia, implying that the Notch pathway was activated in MG63 cells. Additionally, the drug resistance of MG63 cells was significantly reduced through siRNA interference with Notch 1 in hypoxia.^182^ Reducing intracellular drug accumulation is an important approach to regulate tumor drug resistance, and ATP‐binding cassette (ABC)‐mediated drug efflux is associated with multidrug resistance in OS, and MDR‐related protein‐1 (MRP1, also known as ABCC1) is a major target of ABC.^183^ The expression of NICD and PSEN1 (a γ‐secretase catalyst involved in intracellular Notch 1 protein synthesis) significantly increases in OS cells with drug resistance with high ABCC1 expression, and stimulation of the Notch pathway leads to the formation of the NICD/CBF1 complex, which consequently reduces ABCC1 expression, resulting in enhanced drug sensitivity in OS cells.^184^

P53 is the most well‐studied oncogene and is intimately linked to diverse biological behaviors like growth arrest, senescence, apoptosis, and DNA repair.^185^ Patients with Li–Fraumeni syndrome, caused by congenital p53 gene inactivation, exhibit a considerably elevated risk of developing OS as opposed to those with normal p53 genes.^186^ Mutant p53 genes increase the responsiveness of OS‐resistant cells to chemotherapy regimens by upregulating the expression of the proapoptotic genes p21 and Bax.^187^ miR‐34c regulates Notch signaling (HEY2, JAG1, and NOTCH1) and p53‐mediated transcription of several genes implicated in the cell cycle and apoptosis (CCNE2, E2F5, E2F2, and HDAC1).^188^ EMT is a key process of tumor invasion and metastasis.^189^ Su et al.^190^ reported that ATG4A could promote EMT in OS by downregulating Notch 1 and Hes 1 expression and upregulating E‐calmodulin expression through ATG4A knockdown in OS with siRNA, thereby activating the Notch signaling pathway.

### 2.7 MAPK signaling pathway

#### 2.7.1 Overview of MAPK signaling pathway

MAPK is a tightly controlled signaling pathway with Ser/Thr protein kinase activity that performs an instrumental function in diverse biological processes in living organisms, it can be activated by stimuli or cellular signals such as physical stress and inflammatory responses.^191^ The MAPK signaling pathway family primarily encompasses ERK1/2, JNK, p38MAPK, and ERK5.^192^ ERK1/2 has a fundamental function in cell growth, differentiation, and proliferation.^193^ JNK is implicated in cell differentiation, apoptosis, and stress.^194^ p38MAPK is involved in cytokine production, transcriptional regulation, and apoptosis.^195^ ERK5 performs a modulatory role in cell survival, differentiation, proliferation, and other important pathophysiological processes.^196^ Additionally, the MAPK pathway performs a remarkable function in cellular proliferation, angiogenesis, differentiation, apoptosis, and tumor metastasis when compared with the other pathway.^197^ However, the MAPK pathway has been less frequently studied as a major target in OS.

#### 2.7.2 MAPK signaling pathway and OS development and progression

VDAC1 independently functions as a prognostic indicator in OS and patients with high VDAC1 expression exhibit a low survival rate. VDAC1 promotes OS cell proliferation and apoptosis through the MAPK signaling pathway.^198^ Transcriptome analysis showed that ITGB3 functions in OS through the MAPK and VEGF signaling pathways for cell proliferation and cisplatin resistance.^199^ Protein serine kinase H1 (PSKH1) is an autophosphorylated human protein serine kinase. β‐Phenylethyl isothiocyanate induces the proliferation of human OS cells by changing iron metabolism, disrupting redox homeostasis, and activating the MAPK pathway. p38 phosphorylation in OS cells is upregulated by PSKH1, and p38 overexpression promotes the proliferation of OS cells.^200^

### 2.8 NF‐κB signaling pathway

#### 2.8.1 Overview of NF‐κB signaling pathway

NF‐κB belongs to the Rel family of eukaryotic TFs and consists of two subunits: p50 and p65.^201^ Under normal conditions, cytoplasmic NF‐κB undergoes inactivation by binding to the repressor protein IκB in a trimeric complex.^202^ Upon external stimuli such as tumor necrosis factor‐α signals, inflammatory factors, and ultraviolet light, IκB dissociates from the p50/p65/IκB heterodimer, and the NF‐κB dimer subsequently exposes nuclear localization sequences, thereby rapidly translocating from the cytoplasm to the nucleus to bind to specified intranuclear DNA sequences and to trigger or enhance the transcription of associated genes.^203^ Downstream genes of NF‐κB include cyclin D1 and c‐Myc, whose sustained activation may enhance cell growth and contribute to uncontrolled cell proliferation.^204^

#### 2.8.2 NF‐κB signaling pathway and OS development and progression

In OS, the NF‐κB pathway performs an indispensable function in modulating tumor growth, metastasis, and chemoresistance. Aberrant expression of NF‐κB is closely associated with OS. Li et al.^205^ reported the inhibition of OS cell metastasis by combining the blockade of integrin β1 expression and NF‐κB signaling in MG63 cells. Notably, alpha thalassemia and intellectual disability syndrome X‐linked (ATRX) deficiency promotes OS cell invasion by increasing NF‐κB signaling and integrin β3 binding.^206^ The oncogene TRIM10 upregulates nuclear levels of p65, thereby activating classical NF‐κB signaling to promote cisplatin resistance in OS cells.^207^

Contemporary research on NF‐κB‐associated mechanisms in OS is centered on the modulation of miRNAs and lncRNAs that are possible upstream modulators of NF‐κB and could exert their oncogenic or their inhibitory properties on tumors by affecting the levels of STAT3 expressed in OS cells.^208^, ^209^ When the expression of lncRNA NKILA was detected in 60 cases of OS and adjacent tissues, lncRNA NKILA expression was found to be reduced in OS tissues, and the proliferation, invasive rate, and migration of OS cells were enhanced after transfection of NKILA‐siRNA with cells, while the NF‐κB inhibitor (JSH) could reverse the inhibitory impact of NKILA on cell migration and proliferation, implying that lncRNA NKILA is involved in OS development and acts through the NF‐κB/NAIL signaling pathway.^210^ Nectin‐4, an oncogene, can activate the PI3K/AKT/NF‐κB signaling pathway by downregulating miR‐520c‐3p expression, thereby promoting OS progression and metastasis.^211^

## 3 MECHANISM OF TARGETING SIGNALING PATHWAYS

The current treatment for patients with OS is mainly neoadjuvant chemotherapy, including high‐dose methotrexate, doxorubicin, and cisplatin, and surgical excision of the lesion with adjuvant chemotherapy, but the treatment for OS is still slightly inadequate compared with the advantages of chemotherapy combined with targeted therapy for lung cancer and breast cancer. This part aims to discuss the molecular biological pathogenesis of OS, from both small molecule inhibitors and TCM, which are strongly correlated with the onset and advancement of OS, and to explore the corresponding therapeutic targets and downstream pathways to provide strong evidence for further research.

### 3.1 Small molecule inhibitors

A class of organic compounds with molecular weights less than 1000 Da known as small molecule inhibitors can target proteins, reduce protein activity or block biochemical reactions, and are widely used in the study of signaling pathways.^212^ Most of the drugs used in clinical practice have small‐molecule inhibitors as their main components. Extensive research has validated the integral function of small molecule inhibitors in OS.

#### 3.1.1 PI3K/AKT/mTOR signaling pathway

LY294002, the first broad‐spectrum PI3K inhibitor, has a broad inhibitory effect on PI3K‐related pathways in OS cells, and it is often used in combination treatment with other oncogenic drugs in clinical studies to improve drug efficacy and to decelerate the developed drug resistance.^48^ Preliminary data from a phase 2 trial showed that the pan‐I PI3K inhibitor BKM120 was used as a second‐line therapeutic agent for OS, and BKM120‐treated OS cells showed responses in the form of cell proliferation arrest, increased cellular apoptotic rate, and upregulated caspase‐3 activity.^213^ In addition, PI3K inhibitors can reverse anlotinib resistance in OS and restrict OS cell development when used in combination with anrotinib.^214^ Further studies on the effects of downstream molecules on PI3K inhibitors in OS are needed. In 143B cells, ZIP10 overexpression increased protein expression levels of p‐AKT and promoted tumor growth and chemoresistance, while this promotion was attenuated by the AKT inhibitor GSK690693.^215^ Western blotting results revealed that 2‐methylbenzoyl berbamine inhibited the activation of the AKT pathway in OS.^216^ Although a few studies on the use of AKT inhibitors in OS are available, many are only at the cellular experimental stage, necessitating further studies.

With the current development of drugs targeting the mTOR pathway and the limited benefit of monotherapy, multidrug combinations will become the main treatment modality for OS in the future. In human OS cells and mouse OS models in vitro, simultaneous inhibition of PI3K and mTOR targets effectively induced cellular apoptosis, whereas single‐targeted inhibition of PI3K or mTOR was ineffective.^217^ When the dual PI3K/mTOR inhibitor CCT128930 is applied to OS cells lines (MG63, U2OS, and Saos‐2), cyclin D1 and/or cyclin B1 expression was downregulated in tumor cells, and the cell cycle was stalled at the G0/G1 phase.^218^

#### 3.1.2 Wnt/β⁃catenin signaling pathway

The enzyme COX2 celecoxib can inhibit β‐catenin from translocating to the nucleus by activating the GSK3β signaling pathway, decreasing the abnormal activation of the β‐catenin pathway, and inhibiting the growth, invasive, and migratory capacities of OS cells by increasingly suppressing the Wnt/β‐catenin pathway.^219^ TIKI2 functions as an inhibitor protease of the Wnt/β⁃catenin pathway, and its expression is reduced in OS cells.^220^ It can inactivate the Wnt/β‐catenin pathway by inhibiting the downstream GSK‐3β pathway and by removing the N‐terminal amino acid. Restoration of TIKI2 expression through adenoviral transfection promoted phosphorylated GSK‐3β expression and reduced β‐catenin levels within the nucleus, which inhibited the proliferation of OS cells.^221^

Accumulating evidence suggests that microRNA (miRNA) dysregulation and the Wnt/β‐catenin pathway together trigger cancer development, metastasis, and drug resistance.^222^ miRNAs play a targeted role in regulating gene expression, and through gene modification, some miRNA promoters may contain specific binding sites for cellular signaling molecules involved in the Wnt signaling pathway.^223^ These molecules can either activate or inhibit various steps of the Wnt signaling pathway. As for tumor pathogenesis and prognosis, miRNAs act as oncogenes and regulate the expression of genes posttranscriptionally, and they are implicated in OS pathogenesis, indicating a more promising application.^224^ miR‐429 inhibits OS progression by targeting homologous cassette A9 (HOXA9, HOX gene), thereby suppressing tumor cells proliferation, migration, and invasion and attenuating the Wnt/β‐catenin protein signaling pathway.^225^ Yang et al.^226^ reported that miR‐183 targets low‐density lipoprotein receptor‐related protein 6 (LRP6), which regulates the Wnt/β‐connexin signaling pathway and is a downstream effector of LRP6. miR‐183 performs the role of a tumor suppressor miRNA in reducing OS cell migration, invasion, and metastasis.

#### 3.1.3 JAK/ STAT3 signaling pathway

Existing research on hindering OS development by inhibiting the JAK/STAT3 pathway is centered on the inhibitory effects of JAK2 inhibitors and STAT3 inhibitors. AG490, a selective inhibitor of JAK2, targets the JAK signaling pathway in Saos‐2 cells and reduces the proliferative and migratory capacities of OS cells by decreasing the phosphorylation level of STAT3 and impeding its activation, which eliminates the stimulatory effect of THAP9‐AS1 on cellular processes.^227^ In the Saos‐2 model, AG490 inhibits the development of in situ tumors.^117^

Similar to the synthesis‐specific small‐molecule, LLL3,^115^ LLL12,^228^ and FLLL32 reduce the ability of STAT3 to bind to DNA, downregulate the levels of total STAT3 and p‐STAT3, inhibit cell proliferative and migratory capacities, and contribute to caspase 3‐dependent cell apoptosis. The is also a decrease in the protein and mRNA expression levels of STAT3 downstream target genes like VEGF, MMP‐2, and survivin.^229^, ^230^ In OS, apatinib suppresses the OS cell growth, blocks cells in the G0/G1 phase,^231^ and induces apoptosis and autophagy.^232^ NHWD‐870 disrupts the binding affinity of BRD4 to the upstream receptor GP130 promoter region of STAT3, impairs upstream signaling, blocks STAT3 dimerization, and induces apoptosis.^233^

#### 3.1.4 Hippo signaling pathway

In xenograft OS, Verteporfin is known to inhibit YAP/TAZ signaling and suppress the proliferation, migration, and invasiveness of spontaneous OS owing to the deficiency of Rb1 and Trp53 in cells that express Ctsk by blocking the Hippo pathway.^234^ RhoA knockdown significantly reduces the levels of downstream target genes and unphosphorylated YAP, whereas RhoA knockdown remarkably enhanced the apoptosis in OS cells under both in vivo and in vitro conditions after pyropheophorbide‐α methyl ester‐mediated photodynamic therapy.^235^ Fisetin upregulated the levels of leucine zipper‐containing kinase (ZAK) to trigger the Hippo signaling pathway and mediate JNK/ERK stimulation downstream of ZAK, thereby triggering apoptosis in human OS cells in an AP‐1‐dependent manner. Notably, the negatively modulated connective tissue growth factor (CTGF) is targeted by miR‐584‐5p and this regulation results in the inhibition of OS cell metastasis. Moreover, by regulating CTGF in OS, miR‐584‐5p inactivates the Hippo pathway.^236^ As a miR‐34c target, PLOD1 promotes cellular growth and metastasis via the inactivation of the Hippo pathway and the inhibition of LATS1 phosphorylation in OS.^237^

#### 3.1.5 Notch1 signaling pathway

In vivo experiments have shown that DAPT (GSI‐IX), a common synthetic γ‐secretase inhibitor, specifically blocks γ‐secretase and prevents the activation of the Notch signaling pathway.^238^ When DAPT was tested in a mouse para‐tibial tumor model of OS, a K7M2 cell line, and a lung metastasis model, it inhibited ERK pathway phosphorylation by suppressing Notch activation to effectively suppress tumor growth, angiogenesis, and lung metastasis, thereby improving patient survival.^239^ Another γ‐secretase inhibitor, RO4929097, reversed the effects of human umbilical vascular endothelial cell‐derived exosomes on Notch 1, Hes 1, and Hey 1 expression by inhibiting the Notch1 signaling pathway.^240^ A novel DNA methyltransferase 1 (DNMT‐1) small‐molecule antagonist/inhibitor, named DI‐1 (DNMT‐1 inhibitor), enhances the antitumor effects of the molecularly targeted drug cabozantinib in OS cell lines.^241^ Lutetium ethylenediaminetetramethylene phosphonate is a radiopharmaceutical commonly used for its analgesic effect in patients with bone cancer, and relaxin inhibits the Notch‐1 pathway in OS cells through the Notch‐1 pathway.^242^ When IL‐24, an oncogene with cytokine properties cloned from human melanoma HO‐1 cells, was administered via injection into the lateral abdomen of nude mice to treat OS, a substantial reduction in the expression levels of Ki‐67, a biomarker of cellular proliferation, was observed. In addition, treatment of the 143B OS mouse model with IL‐24 revealed inhibition of the tumor growth rate and downregulation of Notch 1, Hes 1, and β‐catenin expression in xenograft tumor tissues, suggesting that IL‐24 may be a new target to improve long‐term survival of patients with OS.^243^

#### 3.1.6 MAPK signaling pathway

In an in situ OS transplantation model, iron chelators induce apoptosis in OS cells owing to their disruption of the homeostatic balance of intracellular iron and activation of the MAPK pathway.^21^, ^244^ Theaflavin‐3,3′‐digallate (TF3) possesses strong antitumor characteristics in the treatment of several cancers affecting humans. In both in vivo and in vitro models, TF3 triggers cellular iron death and apoptosis by activating the ROS and MAPK signaling pathways, reducing glutathione depletion, promoting the accumulation of reactive oxygen species (ROS), and enhancing the Fenton response‐induced oxidative response.^245^ Additionally, the p38 MAPK inhibitor SB203580 effectively blocks the protumorigenic effect of PSKH1.^200^

#### 3.1.7 NF‐κB signaling pathway

The multipotent cytokine macrophage migration inhibitory factor (MIF) inhibitor 4‐iodo‐6‐phenylpyrimidine impedes the binding of MIF with CD74 and p65 and significantly reduces the proliferation and metastasis of OS cells by inhibiting the NF‐κB pathway.^22^

### 3.2 Traditional Chinese medicine

**FIGURE 3:**
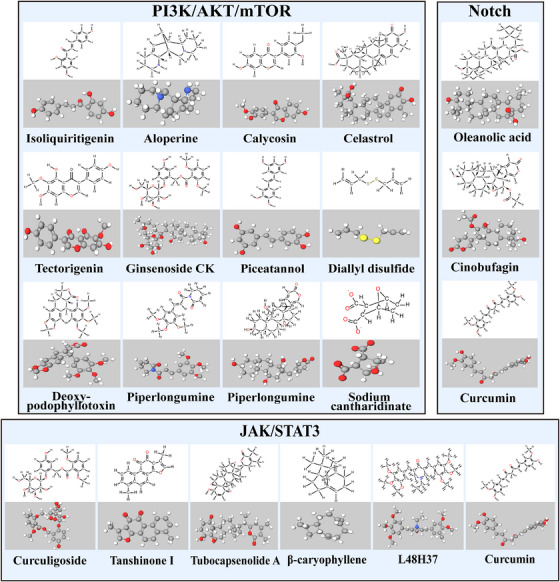
A partial herbal medicine that acts on PI3K/AKT/mTOR, JAK/ STAT3, and NF‐κB signaling pathway in osteosarcoma. Figure are made by Chemdraw. Physakengose G molecular formula is uncertain and not shown in the figure.

**FIGURE 4:**
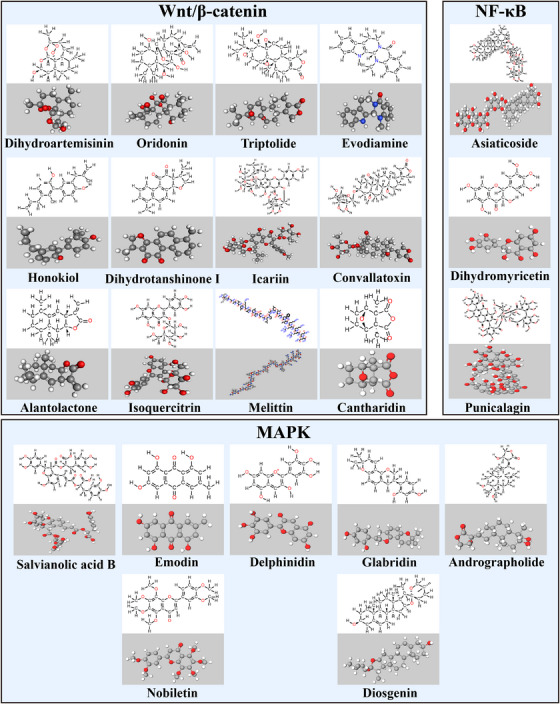
A partial herbal medicine that acts on Wnt/β‐catenin, MAPK signaling pathway in osteosarcoma. Figure are made by Chemdraw. Polyphyllin I and Ganoderma lucidum molecular formula is uncertain and not shown in the figure.

TCM has numerous active monomeric components and their mechanisms of action are complex and diverse. Relying on the synergistic modulation of multiple components, targets, and pathways, TCM plays multiple therapeutic functions in the onset, progression, and metastasis of OS. In recent years, in the study of OS‐targeted signaling pathways, in addition to human‐assembled small molecule inhibitors, more and more studies on natural products have been conducted in recent years (Figures 3 and 4).

#### 3.2.1 PI3K/AKT/mTOR signaling pathway

Sodium cantharidinate is a semisynthetic derivative of cantharidin hydrolyzed by sodium hydroxide.^246^ In MG63 cells, sodium cantharidinate activates the PI3K/AKT pathway, resulting in the activation of p53/p21 and inhibition of the expression levels and activities of cyclin D1 and the protein kinases Cdk‐4 and Cdk‐6, thus leading to the cell block in the G0/G1 phase and suppression of the proliferation of human OS MG63 cells.^247^ In human OS cells, Piperlongumine was shown to trigger apoptosis and G2/M phase blockade by modulating the ROS/PI3K/AKT pathway.^248^ mTOR performs a critical function in the upstream regulation of autophagy, and the PI3K/AKT pathway is the most characteristic regulatory pathway that activates mTOR.^249^ Diallyl disulfide is a natural organic compound extracted from garlic and shallots. Treatment of MG63 cells with diallyl disulfide results in a decrease in PI3K expression and phosphorylation of AKT protein, as well as a decrease in the protein expression of mTOR and its downstream effectors p70S6K and p‐p70S6K. It is suggested that diallyl disulfide blocks the PI3K/AKT/mTOR pathway to induce cellular autophagy.^250^ Deoxypodophyllotoxin, a natural compound from herbal medicine, induces cytoprotective autophagy by downregulating p‐AKT and phosphorylating mammalian target of rapamycin protein (p‐mTOR) in U2OS cells and suppressing the activation of the PI3K/AKT/mTOR pathway.^251^ In human OS cells, the new compound Physakengose G, which was initially extracted from Physalis alkekengi var, triggers apoptosis via EGFR/mTOR signaling and blocks autophagic flux.^252^

Bcl‐2 is a major target molecule for studying the molecular mechanisms of apoptosis. The members of the Bcl‐2 protein family encompass proapoptotic proteins (Bax, Bak, and Bid) and antiapoptotic proteins (Bcl‐xL and Bcl‐2), which control the release of mitochondria‐associated apoptotic factors by controlling the mitochondrial outer membrane permeability. Aloperine,^253^ piceatannol,^254^ calycosin,^255^ and tectorigenin,^256^ all increase the levels of caspase‐3 and Bax, downregulate the levels of PI3K, Bcl‐2, and p‐AKT1, and induce apoptosis in OS cells via the PI3K/AKT/mTOR pathway. Upregulation of MMPs can lead to degradation of the basement membrane and extracellular matrix. The invasion‐related proteins MMP‐2 and MMP‐9 are regulated by AKT.^257^ Ginsenoside CK downregulates the levels of p70S6K1, MMP2, and MMP9 in MG63 and U2OS cells and plays a synergistic role in cell migration and invasion. It is hypothesized that ginsenoside CK suppresses the viability and proliferation of OS cells, induces apoptosis, and reduces cell migratory and invasive capacities by blocking the PI3K/mTOR/p70S6K1 pathway.^258^ Other active ingredients of TCM, such as gamabufotalin,^259^ celastrol,^260^ and isoliquiritigenin,^261^ can reduce the MMP‐2 and MMP‐9 expression levels via the PI3K/AKT pathway, thereby inhibiting OS metastasis.

#### 3.2.2 Wnt/β‐catenin signaling pathway

GSK‐3β can phosphorylate β‐catenin at four N‐terminal sites and is thus a marker of β‐catenin phosphorylation. Following phosphorylation, β‐catenin is degraded by the proteasome through ubiquitination.^262^ Dihydroartemisinin inhibits the growth of OS cells and nude mice transplanted tumors by inhibiting the phosphorylation of serine 9 (Ser9) in GSK‐3β, which activates GSK‐3β to degrade β‐catenin. At the same time, artemisinin downregulates the Dvl protein in OS cells, which unregulates the effect of the Dvl protein on β‐catenin growth by GSK‐3β. At the same time, dihydroartemisinin downregulated Dvl protein expression in OS cells, dysregulated GSK‐3β activity, a key component of the degradation complex formed by GSK‐3β, APC, and Axin, and degraded β‐catenin through GSK‐3β phosphorylation. Inactivation of the Wnt/β‐catenin inhibits OS activity.^263^ Oridonin inhibits the proliferative effect of 143B cells by increasing GSK3β activity, upregulating Dickkopf‐1 (Dkk‐1) expression, and downregulating β‐catenin expression. Moreover, Dkk‐1 overexpression or β‐catenin knockdown enhanced the inhibitory impact of oridonin on the proliferation of 143B cells.^264^ Cantharidin,^265^ triptolide,^266^, ^267^ polyphyllin I,^268^ piperidin,^269^ and evodiamin^270^ can inhibit GSK‐3β phosphorylation and β‐catenin activity, downregulate the Snail, vimentin, N‐cadherin, MMP‐2, MMP‐7, and MMP‐9 expression and upregulate the levels of E‐cadherin, thereby effectively inhibiting OS cell proliferation and metastasis as well as ectopic angiogenesis by regulating the Wnt/β‐catenin pathway.

In vitro trials have illustrated that oleuropein inhibits the proliferation and invasion of OS cells, promotes apoptosis, and exerts good anticancer effects. Additionally, it reduces the expression of the cytoplasmic total β‐catenin in OS cells, reduces the translocation of β‐catenin into the nucleus, and blocks the Wnt/β‐catenin pathway. Notably, the reduction of Wnt‐specific TOP/FOP luciferin reporter gene expression indicated an attenuated transcriptional activity of the TF TCF/LEF in the Wnt/β‐catenin pathway. Furthermore, the expression of the Wnt target genes MMP2 and MMP9 was downregulated to inhibit the migratory invasive ability of cells.^271^ Isoquercitrin remarkably suppressed the proliferative rate, triggered EMT‐associated migratory and invasive activity, and promoted apoptosis in OS cells in vitro. Moreover, β‐catenin together with its downstream genes (survivin, cyclin D1, and c‐Myc) were remarkably downregulated.^272^ Tie et al.^273^ showed that icariin induced apoptosis and inhibited the growth of OS cells by downregulating the Wnt/β‐catenin pathway and attenuating the expression of TOPFlash fluorescence. Conversely, TOPFlash fluorescence expression and adenovirus overexpression of β‐catenin reversed the inhibitory impact of icariin. Ganoderma lucidum,^274^ melittin,^275^ and dihydrotanshinone I^276^ inhibit the Wnt coreceptor LRP5/6 and the Wnt‐associated target genes MMP‐2, MMP‐9, C‐Myc, β‐catenin, and cyclin D1 to block Wnt/β‐catenin signaling and inhibit the EMT process in OS cells, consequently suppressing OS cell proliferation and metastasis. Convallatoxin^277^ and alantolactone^278^ can inhibit the malignant OS progression by blocking the Wnt/β‐catenin pathway.

#### 3.2.3 JAK/STAT signaling pathway

Besides the human‐assembled small‐molecule inhibitors, natural products have recently gained increasing attention, and their extracts have monomeric components that can inhibit JAK/STAT pathway. In vivo studies suggest that curcumin remarkably and dosage‐dependently lowers the levels of p‐JAK2 and p‐STAT3 expressed in MG63 cells. Also, curcumin suppresses the proliferative, migratory, and invasive capacities of MG63 cells, induces G0/G1 phase arrest, and promotes apoptosis through the inhibition of the p‐JAK2/p‐STAT3 pathway.^279^ The curcumin analog L48H37 lowered the phosphorylation level of JAK3, STAT3, JAK2, and JAK1 in U2OS cells, thereby inhibiting the invasion and migration of these cells.^280^ Tubocapsenolide A inhibits OS cell proliferation through SHP‐2‐induced JAK/STAT3 pathway inactivation.^105^ β‐Caryophyllene regulates the JAK1/STAT3 signaling pathway in MG63 cells through the ROS‐induced apoptotic DNA fragmentation mitochondrial pathway, thereby promoting OS cells apoptosis and inflammation.^281^ In vivo and in vitro models revealed that both Tanshinone I,^282^ an extract of Danshen, and curculigoside,^17^ a natural component of *Curculigo orchioides* Gaertn, suppressed the growth and metastasis of OS cells by inhibiting the JAK/STAT3 pathway.

#### 3.2.4 Notch signaling pathway

A thorough analysis of the function played by curcumin in OS revealed that Notch 1/2/3 gene signaling was activated in most of the OS cell lines and that the proliferation and metastasis of OS cells were suppressed when Notch 1 signaling and downstream gene expression were downregulated.^283^ Oleanolic acid can dosage‐dependently suppress the proliferative capacity of OS cells while suppressing the activation of the Notch signaling pathway.^284^ Cisplatin (DDP) is a promising anticancer drug, and cinobufagin combined with low‐dose DDP significantly inhibits OS cells activity and suppresses tumor growth and metastasis, thus increasing survival duration in an OS xenograft model of nude mice, while downregulating Notch 1, Hes 1, Hes 5, and Hey‐L mRNA expression.^285^

#### 3.2.5 MAPK signaling pathway

Notably, MAPK pathway proteins play a central function in regulating the expression of MMPs.^286^ Emodin, an anthraquinone compound extracted from Polygonum multiflorum and Aloe vera, can suppress the proliferative capacity of OS cells by inhibiting the activity of the ERK signaling pathway and may promote the apoptosis of OS cells by increasing the caspase‐3 and caspase‐9 expression levels.^287^ Salvianolic acid B increases the levels of cleaved caspase‐3, phosphorylated p38MAPK (p‐p38MAPK), and phosphorylated p53 (p‐p53) expressed in MG63 cells, suppresses OS cellular proliferation, and triggers apoptosis. In addition, salvianolic acid B increases the level of ROS production, and silencing of p38 expression inhibits the anticancer action of salvianolic acid B on the proliferation level, p‐p53 protein expression, and ROS production level of MG63 cells.^288^ Delphinidin reduces the phosphorylation levels of ERK1/2 and p38 MAPK, inhibits the N‐cadherin expression, and promotes the E‐cadherin expression, thereby inhibiting the migration of OS cells, and these effects of delphinidin are enhanced when it is combined with inhibitors of ERK1/2 and p38.^289^

The MAPK pathway is regulated by ERK, JNK, and p38 MAPK kinase, which are activated and phosphorylated to regulate downstream MMP gene expression, subsequently promoting the occurrence and progression of OS.^290^ Nobiletin^291^ suppresses the metastasis of human OS cells upon blocking the expression of ERK and JNK‐mediated MMPs, and Glabridin^292^ achieved the same effect by blocking p38 and JNK‐mediated MMPs. Andrographolide causes arrest in the G2/M phase and induces apoptosis by regulating the ROS/JNK signaling pathway in OS cells.^293^ Diosgenin induces apoptosis in OS cells by upregulating the ROS‐mediated p38 MAPK signaling pathway.^294^

#### 3.2.6 NF‐κB signaling pathway

Punicalagin can interfere with 19 human OS cell lines, including U2OS, MG63, and Saos2. It significantly reduces proliferation and increases apoptosis of OS cells while downregulating IL‐6 and IL‐8 expression levels.^295^ As a critical transcriptional modulator of metastatic‐associated genes, aprepitant decreased the expression levels of angiogenic factors, VEGF proteins, and NF‐κB and suppressed the migratory phenotype of OS cells.^296^ Dihydromyricetin suppresses the metastasis of human OS cells via the inhibition of SP‐1 and NF‐κB‐regulated urokinase fibrinogen activator.^297^ Asiaticoside reverses the malignant characteristics of OS cells induced by the polarization of the M2 phenotype macrophages through the inhibition of TRAF6/NF‐κB.^298^

## 4 ADVANCES IN CLINICAL RESEARCH ON TARGETED THERAPY AND IMMUNOTHERAPY FOR OS

**FIGURE 5:**
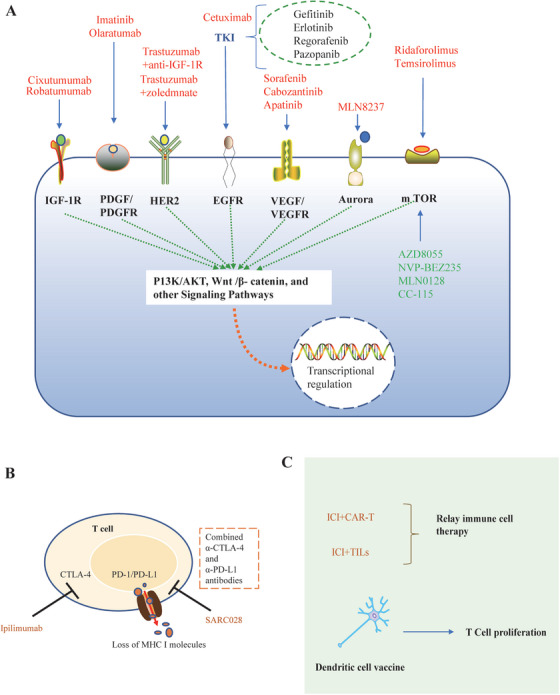
Overview of clinical studies on targeted therapies and immunotherapy for osteosarcoma. The representative therapeutic targets in OS and the corresponding targeted or immunotherapeutic agents that have entered clinical investigations are depicted. (A) Describe targeted therapies in osteosarcoma, (B) describe the immune checkpoint pathways linked to immunity in osteosarcoma, and (C) describe the relay immune cell therapy and tumor vaccines.

Presently, immunotherapy and targeted therapies are the primary focus of the efforts to develop novel treatment regimens for OS. While mounting evidence on cellular and molecular levels indicated that numerous distinct signaling pathways and genes are implicated in the onset and advancement of OS, the druggable fraction of these markers is extremely limited. The principal drug targets for existing drugs include insulin‐like growth factor (IGF)‐R, EGFR, PDGF/PDGFR, mTOR, VEGFR, and Aurora kinase pathways linked to cellular growth, immune checkpoint pathways linked to immunity, relay immune cell therapy, and tumor vaccines (Figure 5).

### 4.1 Targeted therapies

#### 4.1.1 IGF‐R targeted therapy

The IGF system includes three main ligands (IGF‐1, IGF‐2, and insulin), three corresponding tyrosine kinase receptors (IGF‐1R, IGF‐2R, and insulin receptor [IR]) and six distinct IGF‐binding proteins (IGF‐BPs1‐6). They promote cell growth and antiapoptosis, mainly through the MAPK pathway and PI3K/protein kinase B pathway, thereby promoting tumor development and progression. Overexpression of IGF1R has been shown in canine OS and is strongly linked to tumor staging and dismal outcome.^299^ Therefore, many academic research groups and companies have developed neutralizing antibodies (anti‐IGF1R), small molecule tyrosine kinase inhibitors (TKI), or si‐RNA to target IGF1R as a molecular therapeutic strategy.^300^, ^301^, ^302^, ^303^ In a study by WANG et al.,^304^ IGF‐1R was found to be overexpressed in OS tissues, and this overexpression closely affected the tumor metastasis and OS patients’ prognosis. This study also illustrated that attenuating IGF‐1R overexpression could inhibit the growth of OS cells, promote their apoptosis, and enhance the cells to radiotherapy. Consequently, suppression of the IGF system can block its corresponding pathways of action, thus achieving precision therapy.

Cixutumumab (CIX) is an anti‐IGF‐1R monoclonal antibody that functions by binding to IGF‐1R with strong affinity, thereby blocking the interface between IGF‐1R and its ligand and inducing internalization and degradation of IGF‐1R.^305^ Children with refractory solid tumors in phase II trials in 15% of patients with long‐term stable disease showed high tolerance to CIX. When CIX was combined with temsirolimus to treat patients with sarcoma, a remarkable clinical activity was observed; however, no objective responses were identified in phase II trials involving young adults and children with refractory or recurrent sarcomas.^306^ Robatumumab (19D12; MK‐7454, often referred to as SCH717454) is an antibody that is only found in humans and whose function involves binding to and inhibiting IGF‐1R. Three of 31 patients with low disease burden had a complete or partial response, according to solid tumor response assessment criteria.^307^

Human epidermal growth factor receptor 2 (HER‐2) is a tyrosine kinase receptor and a member of the human epidermal growth factor receptor (HER/EGFR/ERBB) family. HER‐2 is remarkably correlated with tumor growth, and targeted therapies for HER‐2 have been used in some solid tumors.^308^ Trastuzumab, an HER‐2‐specific recombinant monoclonal antibody, is currently used in clinical practice to treat patients with HER‐2‐positive breast cancer. Nevertheless, some studies have shown that in patients with HER‐2‐positive OS, trastuzumab combined with chemotherapy did not significantly improve overall survival compared with patients with HER‐2‐negative chemotherapy.^309^ However, other studies have shown that when combined with recombinant human IGF‐1R monoclonal antibodies (anti‐IGF‐1R)^310^ or zoledronic acid (zoledmnate).^311^ They can kill OS cells and significantly increase the growth inhibition rate of HER‐2‐positive OS cells.

Multitarget TKI of EGFR are the fastest advancing class of drugs for targeted therapy of OS. Its mechanism of action is to inhibit the autophosphorylation of intracellular zone receptor tyrosine kinase or competitively suppress the binding of receptor tyrosine kinase ATP to the EGFR site, blocking downstream signaling and is primarily used in the clinical management of advanced non‐small cell lung cancer (NSCLC). Studies on OS have shown that gefitinib significantly reduces the survival of OS cells from starvation stress and can be used as an adjuvant agent to enhance the anti‐OS activity of adriamycin and methotrexate.^312^ At the same time, erlotinib can moderately enhance the chemotherapeutic effect of canine OS cells and may be used as a potential EGFR inhibitor to treat some patients with OS.^313^ Pazopanib and regorafenib are some of the two drugs that have demonstrated high efficacy in OS therapy in several clinical trials.^314^, ^315^ Noteworthy, TKI combined with chemotherapy has been considered during the presurgical neoadjuvant phase to ameliorate the toxic effects of chemotherapy and reduced the duration of therapy. Meanwhile, applicable clinical trials are underway.^316^, ^317^

#### 4.1.2 EGFR targeted therapy

The EGFR family belongs to type I transmembrane tyrosine kinase receptors, including EGFR/ErbBl/Her‐1, ErbB2/Her‐2, ErbB3/Her‐3, and ErbB4/Her‐4. The EGFR also performs a fundamental function in numerous types of cancers. In NSCLC, the driver mutations consist of EGFR mutations, particularly exon 20 insertions and point mutations.^318^, ^319^ Nevertheless, in other types of cancers such as OS, these EGFR mutations are extremely rare and it is not yet elucidated whether they are significant in clinical settings. Mantovani et al.^313^ suggested that activation of the EGFR‐mediated progrowth signaling pathway performs a positive regulatory function in OS onset and advancement. Currently, extensive studies have been conducted on the relevance of EGFR to tumors and have shown positive expression in various malignancies, like lung, breast, cervical, and gastric cancers.^318^ Trials have been conducted in patients with OS, but there is still a need for a large amount of research data.

Cetuximab, a monoclonal antibody against EGFR, blocks EGFR pathway signaling mainly by competitively suppressing the binding action of EGFR to related ligands, which can kill EGFR‐positive OS cells by enhancing the cytolytic function of NK cells and inhibit distant tumor invasion and angiogenesis.^319^

#### 4.1.3 PDGF/PDGFR targeted therapy

PDGF/PDGFR is a significant signaling pathway with remarkable involvement in the proliferation and differentiation of osteoblasts and osteoclasts. Additionally, PDGF/PDGFR performs a central function in the process of tumor angiogenesis by activating downstream signaling molecules through the formation of dimeric complexes by PDGF and PDGFR binding to trigger receptors.^320^ Bozzi et al.^321^ concluded that PDGFR overexpression was associated with disease progression and poor prognosis. There is still disagreement regarding the relevance of PDGF‐A expression to the prognosis of OS. Takagi et al.^322^ showed that in vivo contact between OS and platelets can result in the aggregation of platelets and stimulation of the PDGF secretion, thereby phosphorylating PDGFR and Akt and promoting the proliferative rate of OS cells. Therefore, preventing PDGF from being bound by ligands can inhibit tumor angiogenesis and thus achieve antitumor effects.

Imatinib is a potent TKI that functions by targeting PDGFR signaling and was originally used to treat chronic myeloid leukemia.^323^ Its targets include ABL, PDGFR, and c‐kit. To clarify the inhibitory effect of the PDGF pathway, studies have tested the expression of its downstream signaling proteins and observed that PDGFR phosphorylation and associated downstream signaling molecules were suppressed in >50% of patients after treatment with imatinib. Gobin et al.^324^ showed that imatinib mesylate inhibited tumor growth in an animal model of OS in mice. Meanwhile, in two open‐label, single‐arm phase II clinical trials, the effectiveness of imatinib as a monotherapy drug in patients with OS was shown to be unsatisfactory,^325^ and its use alone is inappropriate for the treatment of advanced OS, but it is reassuring that a synergistic antiproliferative effect of imatinib in combination with adriamycin for OS has been found in ex vivo experiments.^326^ Similar to imatinib, two‐phase I studies of another antibody, olaratumab, evaluated the unsatisfactory efficacy of olaratumab as a single agent in patients with advanced sarcoma.^327^, ^328^, ^329^ However, a phase II study illustrated that when used in tandem with doxorubicin, olaratumab was surprisingly effective as an “add‐on” drug,^330^ and the use of olaratumab in conjunction with doxorubicin resulted in a 48% reduction in the mortality risk in contrast with the use of doxorubicin monotherapy.

#### 4.1.4 mTOR targeted therapy

mTOR is a downstream regulator of PI3K, which is implicated in multiple regulatory functions like cell survival, proliferation, metastasis, and vascular regeneration. Studies have confirmed that mTOR signaling is aberrantly activated in OS and its inhibitors can exert antitumor effects by inhibiting cell growth and proliferation.^331^ Additionally, the combination of mTOR inhibitors with other forms of drugs, like antiosteoporosis drugs, conventional chemotherapeutic drugs, and super‐terminal domain protein inhibitors enhances their antitumor effects.^332^ Another study found that a dual mTOR inhibitor could enhance its antitumor activity in OS via MEK/ERK inhibitors. By blocking the mTOR transduction pathway, it can be used to inhibit the development of OS.^333^

Ridaforolimus rapamycin and its analogs are first‐generation inhibitors of mTOR, inhibiting mTORC1 kinase activity by binding to FKBP‐1.^334^ The results of preclinical testing illustrated the broad antitumor activity of rapamycin alone in OS xenograft models and in vivo. In addition, administering rapamycin in conjunction with vincristine or cyclophosphamide increased the responsiveness in OS models.^335^ Temsirolimus, an analog of rapamycin, exhibited excellent antitumor activity in OS models when combined with cisplatin or bevacizumab.^336^ Ridaforolimus, which is a selective mTOR antagonist, was administered as a monotherapy regimen in a phase II trial to treat patients with advanced sarcoma and it was found that these patients experienced partial symptom relief. Moreover, a larger double‐blind phase III trial illustrated that in patients with metastatic sarcoma, administering Ridaforolimus prolonged progression‐free survival (PFS) compared with chemotherapy.^337^

Everolimus, a 40‐O‐(2‐hydroxyethyl)‐rapamycin derivative, impairs the proliferative potential of OS cells by stopping the G1 phase of the cell cycle. A phase II clinical trial illustrated that the proportion of patients with high‐grade OS treated with sorafenib in conjunction with everolimus increased from 27 to 45% of patients with a median PFS duration of 6 months compared with sorafenib alone, thus showing that the survival benefit of sorafenib combined with everolimus for high‐grade OS was superior to sorafenib monotherapy.^338^ This effect was also confirmed by the experiments of Higuchi et al.^339^

In OS xenograft animals, second‐generation dual mTOR inhibitors with weak antitumor activity included AZD8055, which inhibits both mTOR1 and mTOR2. Nonetheless, additional new small molecules inhibitors, like dual PI3K/mTOR antagonists (NVP‐BEZ235, MLN0128) and dual mTOR/DNA‐PK inhibitors (CC‐115), are still being studied in clinical trials to determine their efficacy.^340^, ^341^

#### 4.1.5 VEGF/VEGFR targeted therapy

Angiogenesis is primarily modulated by VEGF/VEGFR signaling,^342^ and there have been attempts to block angiogenesis as part of therapy for OS. Strategies for blocking angiogenic signals include neutralizing VEGF with antibodies, blocking VEGF receptors with antibodies, and inhibiting the intracellular activity of VEGF with small‐molecule TKIs VEGF targeting in OS has, however, not been effective. Bevacizumab, a monoclonal anti‐VEGF antibody, was evaluated in phase II trials as a first‐line treatment for advanced OS. Bevacizumab in conjunction with chemotherapy did not improve OS; however, treatment with bevacizumab was linked to an increase in PFS and an increase in total remission rates.^343^

Sorafenib is a specific TKI that targets the VEGFR pathway. Heymann et al.^344^ showed that in the OS model, sorafenib is anticipated to limit lung metastasis and attenuate tumor development. A phase II trial showed that sorafenib in patients with OS demonstrated activity as second‐ or third‐line therapy at 4‐month PFS and was the first targeted agent to show activity in patients with OS.^345^ The National Comprehensive Cancer Network guidelines now list sorafenib as a second‐line therapy alternative for advanced OS.

Cabozantinib, a VEGFR2 and MET inhibitor, was evaluated in a phase II clinical trial involving 43 patients with OS, 39 of whom had pulmonary metastases, the 6‐month PFS rate was 33% and only five patients had partial responses, a finding similar to that of the sorafenib trial.^346^ Apatinib is a new, highly selective oral small‐molecule antiangiogenic drug that binds to and blocks VEGFR‐2, vasculogenesis, and tumor growth.^347^ Currently approved for third or more lines of treatment for advanced gastric adenocarcinoma or adenocarcinoma of the gastroesophageal junction, clinical trials of apatinib for OS are now underway.^348^

#### 4.1.6 Aurora kinase targeted therapy

The hallmark of tumor cells is the loss of cell cycle regulation, resulting in uncontrolled cell proliferation. Aurora kinase has functions such as regulating cell mitosis, and abnormal expression or activation of aurora kinase results in the transformation of normal cells into tumor cells.^349^ The development of many Aurora kinase inhibitors has reached various stages. The Aurora kinase A inhibitor, alisertib (MLN8237), specifically targets AURK‐A by competitive binding to ATP.^350^ In human OS U‐2 OS and MG63 cells, it has been shown to exert proapoptotic and proautophagic activities by stimulating the mitochondria‐induced pathway and suppressing the p38 MAPK/PI3K/Akt/mTOR signaling pathway.^351^

### 4.2 Immunotherapy

Over the past 10 years, immunotherapy has revolutionized the way that cancer is treated, and immunotherapy for OS has developed at a breakneck pace. Major cancer immunotherapies include peripatetic immune cell therapy, checkpoint inhibitors, and cancer vaccines, among others. Several solid tumor types can be treated with checkpoint inhibitors. Clinical investigation of other adaptive immune cell therapies and cancer vaccines in solid tumors is ongoing.

#### 4.2.1 Immune checkpoint inhibitors

Researchers have recently developed monoclonal antibodies that are directed against CTLA‐4 and PD‐1/PD‐L1, and they have improved effectiveness in treating malignancies like melanoma^352^ and lung cancer.^353^ PD‐1/PD‐L1 as an immunotherapeutic target has opened a new era of immunotherapy and accelerated the research on OS treatment. Different immunocytes including CTL, NK cells, B cells, and so on, express PD‐1 on their surfaces. PD‐1 can bind to PD‐L1 indicated by cancer cells to deliver programmed death signals to immune cells. In a mouse model of metastatic OS, significant improvement in OS responsiveness to CTL by using antibodies to block PD‐1/PD‐L1 interaction has been observed to contribute to decreased tumor load and improved patient survival.^354^ In addition, blocking the PD‐1/PD‐L1 axis enhances the chemotherapeutic effect of cisplatin in OS.^355^ The effectiveness of these studies was only at the animal model stage, and the relevant clinical studies of monoclonal antibodies against PD‐1/PD‐L1 did not show the same therapeutic effect as the animal models. Le Cesne et al.^356^ conducted a phase II clinical study involving 17 patients with advanced OS and found a 6‐month PFS rate of only 13.3%.

A phase II clinical trial of camrelizumab in conjunction with apatinib for advanced OS also failed to meet the 6‐month PFS preset goal.^357^ A multicenter phase II clinical trial on the effectiveness of pembrolizumab (SARC028) was the first prospective study of the treatment of advanced soft tissue sarcoma and OS by blocking immune checkpoints. Although the study showed some promise for application, the efficacy of the enrolled patients fell far short of expectations. Only 18% of soft tissue sarcoma patients showed a clinical response to pembrolizumab, while only 5% of OS patients showed a clinical response.^358^ Research indicates that PD‐L1 expression levels in OS are variable and usually accompanied by the loss of MHC class I molecules leading to immune escape, which may be an important reason for the poor effectiveness of ICIs in OS.^359^ Therefore, testing the levels of PD‐L1 and MHC class I molecules expressed in patients with OS before ICI therapy may be important to improve the outcome.

Another important checkpoint is CTLA‐4, a transmembrane glycoprotein receptor expressed in regulatory T cells and memory T cells that inhibits antitumor immune response by interacting with CD80/86 on DCs.^360^ Upregulation of T‐cell CTLA‐4 expression levels was found in the blood of 19 patients with OS relative to 16 healthy volunteers, which was the basis for the development of CTLA‐4 inhibitors.^361^ Ipilimumab, a CTLA‐4 monoclonal antibody developed in 2011, was approved by the United States Food and Drug Administration as the first next‐generation immune checkpoint inhibitor for the treatment of melanoma.^362^ A phase I clinical trial study illustrated that 25% of patients with OS had stable disease after treatment with Iplimma.^363^ Combined α‐PD‐L1 and α‐CTLA‐4 antibody blockade immunotherapy performed in a metastatic OS K7M2 mouse model resulted in complete control of most mouse tumors,^364^ which offers a novel approach for treating OS.

#### 4.2.2 Relay immune cell therapy

Relay immune cell therapy is another area of immunotherapy that is rapidly evolving, and CAR‐T therapy is at the heart of this. CAR‐T cells are highly specific and targeted for cytotoxicity, using the immune system's capacity to track down, spot, and eliminate cancerous cells.^365^ CAR‐T cell therapy has achieved good efficacy in malignant blood diseases,^366^ but the poor efficacy in solid cancers is mainly related to the TME, off‐target toxicity, immune escape, and tumor Ag heterogeneity.^367^ In preclinical investigations, CAR‐T cells exhibited substantial antitumor efficacy. The combo of ICI may improve the targeted toxicity of CAR‐T cells, and appropriate target Ags including HER2, GD2, and B7‐H3 can enhance their activity and lessen side effects.^368^ Nonetheless, because OS is a low‐immunogenic and extremely heterogeneous tumor, it is challenging to choose a precise and secure target.

TIL is another form of passaged cell therapy, and studies have shown satisfactory objective response rates of 40 to 70% in patients with metastatic melanoma to TIL.^369^ A preclinical study reported that TILs were detectable in 75% of patients with OS and included CD8+ and CD4+ T‐lymphocytes, CD117 mast cells, and CD20+ B lymphocytes.^370^ The advantage of TILs is that they target tumor‐specific Ags and are highly specific for tumors. WANG et al.^371^ reported that combining TILs with ICIs was clinically effective in treating metastatic OS, with an efficacy rate nearly five times that of a single ICI, and prolonged PFS and overall survival.

#### 4.2.3 Tumor vaccine

Tumor vaccines are used to achieve antitumor effects by inducing a strong immune response against tumors. A widely used tumor vaccine is the DC vaccine, which is an APC that induces the proliferation of cytotoxic T lymphocytes to achieve antitumor effects.^372^ DC vaccines are most commonly used in OS.^373^ Although Mackall et al.^374^ showed that the 5‐year survival rate was prolonged from 31 to 43% in 30 patients who received the DC vaccine compared to the control group, only two of the 12 OS patients observed by Himoudi et al.^375^ received DC vaccine therapy‐induced specific T‐cell immune responses against the tumor. Miwa et al.^376^ evaluated the clinical response in 35 of 37 patients with OS; only six patients had stable disease and 28 patients showed tumor progression. These studies suggest that tumor vaccines for OS are safe and can activate the immune system to some extent, but the therapeutic effect on OS or whether it will improve patient prognosis in combination with other immunotherapies remains unclear and needs to be supported by more relevant studies.

## 5 CONCLUSION AND PROSPECTS

Currently, the signaling pathways related to the development of OS are increasingly being investigated by humans, whether it is PI3K/AKT/mTOR signaling pathway, Wnt signaling pathway, JAK/ STAT3 signaling pathway, Hippo signaling pathway, Notch signaling pathway, PD‐1/PD‐L1 signaling pathway, MAPK signaling pathway, or NF‐κB signaling pathway (Table 1). Related studies have shown their involvement in the development of OS. The mechanism of OS development is a complex signaling network, and in addition to the above eight signaling pathways, some related pathways have not been elucidated in this paper, which needs to be summarized in more depth. Whether one or more pathways are implicated in the regulation of OS signaling pathways has not yet been better demonstrated, and whether single pathways or multiple pathways combine to dominate regulation requires further study. However, there is no doubt that a thorough examination of the mechanisms behind OS evolution will enable us to unlock the secret.

**TABLE 1:** The roles and functions of signaling pathways in OS and the identified biomarkers as well as potential therapeutic targets.

Signaling pathways	Roles and functions: significant roles in OS	Cellular biological processes	Biomarkers and potential therapeutic targets	References
PI3K/AKT/mTOR signaling pathway	Diagnosis associated with lung metastasis, related to chemotherapy resistance	Proliferation, survival, migration, invasion, metastasis, cell cycle, apoptosis, angiogenesis	PI3K, p‐PI3K, AKT, p‐AKT, mTOR, PTEN, miR‐142, miR‐191‐5p, miR‐524, miR‐9‐5p, miR‐744, lncRNA HULC, lncRNA MSC‐AS1	^48^, ^51^, ^52^, ^53^, ^54^, ^55^, ^61^, ^62^
Wnt/β‐catenin signaling pathway	Diagnosis associated with lung metastasis, related to tumor recurrence and chemotherapy resistance	Proliferation, differentiation, survival, cell cycle, apoptosis, migration, invasion, EMT, angiogenesis	β‐Catenin, ROR2, SDC2, sFRP2, WIF‐1, GSK‐3β, miR‐429, miR‐183	^84^, ^86^, ^87^, ^88^, ^92^, ^225^, ^226^
JAK/ STAT3 signaling pathway	Diagnosis associated with lung metastasis, related to chemotherapy resistance	Proliferation, apoptosis, migration, invasion, metastasis, autophagy, and angiogenesis	JAK, STAT3, P‐STAT3, CyclinD1, Bcl‐2, Mcl‐1, COX‐2, MMP‐2, survivin, lncRNA HOXD‐AS1, miR‐126, miR199a‐3p, miR‐199a‐5p, miR‐125b	^110^, ^111^, ^112^, ^113^, ^114^
Hippo signaling pathway	Diagnosis and prognosis biomarker, related to chemotherapy resistance	Proliferation, differentiation, apoptosis, migration, and invasion	YAP, TAZ, Sox2, TEAD1, USP1, miR‐375, miR‐624‐5p, miR‐584‐5p, miR‐34c	^126^, ^127^, ^128^, ^129^, ^130^, ^131^, ^132^, ^236^, ^237^
PD‐1/PD‐L1 signaling pathway	Prognosis biomarker, related to immunotolerance	Proliferation, survival, metastasis, apoptosis, immune response	PD‐1/PD‐L1, IFN‐γ, miR‐140, miR‐200a, miR‐106a, Lnc00657	^143^, ^144^, ^162^, ^163^, ^164^
Notch signaling pathway	Proliferation, differentiation, survival, migration, invasion, metastasis	Formation, development, survival, proliferation, invasion, and metastasis	Jagged1, Notch1, Notch‐ICN, MRP1, Hes1, E‐cadherin, P53miR‐26a, lncRNA MEG3	^175^, ^176^, ^177^, ^178^, ^179^, ^180^, ^181^, ^182^, ^183^
MAPK signaling pathway	Diagnosis associated with lung metastasis, related to chemotherapy resistance	Proliferation, differentiation, apoptosis, ROS, and tumor metastasis	p38, ERK, JNK, VDAC1, ITGB3, PSKH1, MMPs, caspase‐3, caspase‐9	^198^, ^199^, ^200^
NF‐κB signaling pathway	Related to tumor recurrence and chemotherapy resistance	Growth, proliferation, chemoresistance, invasion, and metastasis	ATRX, p65, TRIM10	^206^, ^207^

The development of pulmonary metastasis from OS is not something anyone wants to foresee, but the highly active and highly metastatic nature of OS cells leads to early metastasis to distant tissues and can lead to a range of malignant symptoms, which may further impact the prognosis and the rate of survival for OS patients. Although the patients’ prognoses in OS have not changed significantly in recent decades, and pulmonary metastasis of OS remains a major challenge to curing OS and improving patient survival, various therapeutic approaches have made some progress and have yielded important clinical benefits. Targeted therapies for OS are safer and more effective than chemotherapy. For OS‐targeted therapy, the development of more potent medications and the identification of biomarkers with higher sensitivity and specificity remain the major priorities (Table 2). With continuous developments in molecular biology and advances in basic research, many in vivo and in vitro experiments have identified not only the great potential of targeted drugs but also new regulatory mechanisms and targets. Recent research results have identified novel diagnostic/prognostic biological markers and possible therapeutic targets, enabling a clearer knowledge of the molecular mechanisms underlying OS onset, progression, metastasis, and drug resistance. Several molecular targets, including PI3K, mTOR, PD‐1/PD‐L1, and others, have advanced to at least phase II clinical investigations. The molecular processes, however, typically involve crosstalk or feedback loops instead of particular signaling pathways. When using a single targeted therapy, bypass is a key factor in treatment resistance. New combination regimens must thus be created and validated promptly.

**TABLE 2:** Clinical trials with potential new molecular targets.

Trial	Phase	Drug	Treatment regimen	Molecular target	Results and conclusions	References
NCT01016015, NCT01614795	II	Cixutumumab	Cixutumumab + temsirolimus	IGF‐R	No objective responses were identified in phase II trials involving young adults and children with refractory or recurrent sarcomas.	^306^
NCT00617890	II	Robatumumab	Robatumumab	IGF‐R	Three of 31 patients with low disease burden had a complete or partial response, according to solid tumor response assessment criteria.	^307^
Cooperative Children's Oncology Group (COG)	II	Trastuzumab	Trastuzumab + anti‐IGF‐1R or zoledmnate	HER‐2	Kill OS cells and significantly increase the growth inhibition rate of HER‐2‐positive OS cells.	^310^, ^311^
Cooperative Children's Oncology Group (COG)	II	Imatinib	Imatinib + adriamycin	PDGF/PDGFR	Synergistic antiproliferative	^325^
NCT01185964	II	Olaratumab	Olaratumab + doxorubicin	PDGF/PDGFR	Reducing the risk of death by 48%	^330^
NCT00112372, NCT00093080	II/III	Ridaforolimus	Ridaforolimus + vincristine or cyclophosphamide	mTOR	OS patient had a PR and prolonged PFS compared with chemotherapy.	^335^, ^337^
NCT02429973	II	Everolimus	Everolimus + sirolimus	mTOR	The proportion of median PFS time up to 6 months in patients with single‐agent sorafenib increased from 27 to 45%.	^345^
NCT00667342	II	Bevacizumab	Bevacizumab	VEGF/VEGFR	Associated with increased progression‐free survival and overall remission rate	^343^
NCT02429973	II	Sorafenib	Everolimus + sirolimus	VEGF/VEGFR	Show activity as a second‐ or third‐line treatment in the 4‐month PFS	^345^
NCT02243605	II	Cabozantinib	Cabozantinib	VEGF/VEGFR	The 6‐month progression‐free survival rate was 33%, with only five patients showing a partial response.	^346^
NCT03359018	II	Camrelizumab	Camrelizumab + apatinib	Immune checkpoint inhibitors	The preset target of PFS at 6 months was not reached.	^357^
NCT02301039, NCT03013127	II	Pembrolizumab	Pembrolizumab	Immune checkpoint inhibitors	Only 5% of the patients with osteosarcoma showed a clinical response.	^358^
NCT01445379	I	Ipilimumab	Ipilimumab	Immune checkpoint inhibitors	Twenty‐five percent of patients with osteosarcoma had stable disease after ipllimma.	^363^
NCT01241162, NCT01803152	I/II	DC vaccines	DC vaccines + decitabine or gemcitabine pretreatment	Dendritic cell peptide vaccines	Primary and metastatic tumor growth inhibition and remodeling of tumor microenvironment with reduced Treg and immunosuppressive cytokines and increased CD8+ T lymphocytes, with small outcome benefits in clinical trials.	^374^, ^376^

There are few relatively satisfactory clinical chemotherapy regimens available, and collaborative multiparty studies are needed to identify a chemotherapy regimen that will improve patient prognosis, while basic, translational, and clinical research teams collaborate, leading to the development of new prognostic markers and new therapeutic targets. No single immunotherapy has been definitively shown to be effective in OS, and the clinical efficacy of a single immunotherapy is inadequate, but there are clear studies demonstrating an immune link between OS progression and immunity, so in‐depth studies of immune mechanisms and the development of new immunotherapies are a priority in immunology research. Pain management is also important, not only to improve symptoms but also to benefit patients physically and mentally and their recovery. As immunotherapy and gene therapy are expected to provide more opportunities and possibilities for the treatment of OS, the combination strategy of multiple therapeutic approaches is a hot topic of current research.

This review also focuses on the signaling targets involved in the preventive and curative effects of TCM on OS through the modulation of related protein and gene expression in signaling pathways. Although the research results of TCM for OS are encouraging, most of the current experimental studies focus on the impact of the drugs on OS cells in the context of inhibiting proliferation or promoting apoptosis, and only a few research reports have specifically examined the impact on metabolism and immunity, which is relatively single‐directional research, and it is currently difficult to achieve clear clinical efficacy. Regarding the action mechanism of TCM in OS therapy, most of the TCM compounds are still under the general framework of multitarget and multipathway synergy, and their specific targets have not been fully elucidated. Given the complex and multitarget components of TCM, more extensive research is required. to realize the synergistic effects of multiple TCM extracts to achieve multitarget, multipathway, and multifaceted treatment of OS. Moreover, future studies should focus on comprehensively revealing the mechanism of action of herbal components and compound formulations from a holistic perspective^.^

## AUTHOR CONTRIBUTION

*Writing—original draft*: Ji Ziyu. *Writing—original draft, visualization, funding acquisition*: Shen Jianlin. *Methodology, writing—review and editing, funding acquisition*: Yi Qian. *Conceptualization, writing—review and editing, supervision, funding acquisition*: Liu Huan. *Draw figure*: Yujian Lan. All authors have read and approved the final manuscript.

## CONFLICT OF INTEREST STATEMENT

The authors declare no conflicts of interest.

## ETHICS STATEMENT

Not applicable.

## Data Availability

Not applicable.
